# Understanding the Role of the Master Regulator XYR1 in *Trichoderma reesei* by Global Transcriptional Analysis

**DOI:** 10.3389/fmicb.2016.00175

**Published:** 2016-02-16

**Authors:** Lilian dos Santos Castro, Renato G. de Paula, Amanda C. C. Antoniêto, Gabriela F. Persinoti, Rafael Silva-Rocha, Roberto N. Silva

**Affiliations:** ^1^Molecular Biotechnology Laboratory, Department of Biochemistry and Immunology, Ribeirão Preto Medical School, University of São PauloRibeirão Preto, Brazil; ^2^Laboratório Nacional de Ciência e Tecnologia do Bioetanol, Centro Nacional de Pesquisa em Energia e MateriaisCampinas, Brazil; ^3^Systems and Synthetic Biology Laboratory, Department of Cell and Molecular Biology, Ribeirão Preto Medical School, University of São PauloRibeirão Preto, Brazil

**Keywords:** *Trichoderma reesei*, RNA-seq, XYR1 transcription factor, gene expression, enzymes

## Abstract

We defined the role of the transcriptional factor—XYR1—in the filamentous fungus *Trichoderma reesei* during cellulosic material degradation. In this regard, we performed a global transcriptome analysis using RNA-Seq of the Δ*xyr1* mutant strain of *T. reesei* compared with the parental strain QM9414 grown in the presence of cellulose, sophorose, and glucose as sole carbon sources. We found that 5885 genes were expressed differentially under the three tested carbon sources. Of these, 322 genes were upregulated in the presence of cellulose, while 367 and 188 were upregulated in sophorose and glucose, respectively. With respect to genes under the direct regulation of XYR1, 30 and 33 are exclusive to cellulose and sophorose, respectively. The most modulated genes in the Δ*xyr1* belong to Carbohydrate-Active Enzymes (CAZymes), transcription factors, and transporters families. Moreover, we highlight the downregulation of transporters belonging to the MFS and ABC transporter families. Of these, MFS members were mostly downregulated in the presence of cellulose. In sophorose and glucose, the expression of these transporters was mainly upregulated. Our results revealed that MFS and ABC transporters could be new players in cellulose degradation and their role was shown to be carbon source-dependent. Our findings contribute to a better understanding of the regulatory mechanisms of XYR1 to control cellulase gene expression in *T. reesei* in the presence of cellulosic material, thereby potentially enhancing its application in several biotechnology fields.

## Introduction

Our dependence on fossil fuels and concerns surrounding its impacts on the environment has generated worldwide interest in establishing new fuel and energy sources (Borin et al., 2015). Reduction in fossil fuel consumption using alternate energy sources is a major challenge that humankind will face in the coming decades. Bioethanol production using lignocellulosic biomass, however, is a viable option for addressing this challenge (Ulaganathan et al., 2015). Lignocellulosic biomass is among the most abundant renewable resources on the planet and is an alternative sustainable energy source for the production of second-generation biofuels. Simple or complex carbohydrates can be extracted from lignocellulosic biomass that is fermented by microorganisms during bioethanol production (Kricka et al., 2015).

Bioconversion of cellulose-containing substrates to glucose represents an important area of modern biotechnology. Enzymes required for degradation of the polysaccharide component of biomass are mostly produced by fungi belonging to the genus *Trichoderma* (Gemishev et al., 2014). The filamentous fungus *Trichoderma reesei* (*Hypocrea jecorina*) is of particular interest due to its remarkable ability to secrete large amounts of cellulases, which include exoglucanases (EC 3.2.1.91) that cleave cellobiose into cellulose strand ends; endoglucanases (EC 3.2.1.4) that cleave strands randomly; and β-glucosidases (EC 3.2.1.21) that convert soluble cellodextrins; and cellobiose to glucose (Song et al., 2015; Treebupachatsakul et al., 2015). An additional protein, swollenin (encoded by *swo1*), can disrupt crystalline cellulose structures, presumably making polysaccharides more accessible to hydrolysis (Saloheimo et al., 2002). Moreover, in fungi, secreted copper-dependent polysaccharide monooxygenases (PMOs), a novel class of enzymes previously termed Glycoside Hydrolases Family 61 (GH61s), boost the efficiency of common cellulases, resulting in enhanced hydrolysis while decreasing the initial protein load needed. GH61s act on the crystalline part of cellulose generating oxidized, and non-oxidized chain ends (Dimarogona et al., 2012; Li et al., 2012) in microorganisms such as in *Chaetomium globosum, Myceliophthora thermophile, Thielavia terrestris, Neurospora crassa, T. reesei*, and *Aspergillus niger* (Glass et al., 2013). Recent studies of GH61 fungal proteins (Quinlan et al., 2011; Beeson et al., 2012; Dimarogona et al., 2012; Horn et al., 2012) have shown that the classical endo/exo scheme indeed may be simple. These proteins possess flat substrate-binding surfaces and are capable of cleaving polysaccharide chains in their crystalline contexts using an oxidative mechanism that depends on the presence of divalent metal ions and an electron donor, such as cellobiose dehydrogenase (CDH), a potential electron donor for PMOs (Dimarogona et al., 2012). A deeper mechanistic understanding of these enzymes could be used to further reduce costs of lignocellulosic biofuel production (Beeson et al., 2012). Consequently, numerous details pertaining to the structure and function of these enzymes have been elucidated, and several aspects of the regulation of their expression and secretion into the medium have also been described (Kubicek, 2012).

The production of these extracellular enzymes is an energy-consuming process; therefore, enzymes are produced only under conditions where the fungus needs to use plant polymers as energy and carbon sources (Amore et al., 2013). To metabolize cellulose, cellulase production is regulated at the transcriptional level in a coordinated manner that depends on the availability of the carbon sources (Dashtban et al., 2011; Amore et al., 2013; Castro Ldos et al., 2014). Pure (oligo)saccharides, such as sophorose (Sternberg and Mandels, 1979), β-cellobiono-1,5-lactone, D-xylose, xylobiose, galactose, and lactose, have been also reported to induce cellulase and hemicellulase production in *T. reesei* (Sternberg and Mandels, 1979; Aro et al., 2005; Stricker et al., 2007; Hakkinen et al., 2014; Tani et al., 2014). Various environmental and physiological factors affect enzyme production and secretion in *T. reesei*, suggesting that a complex signaling cascade and regulatory network is required for the accurate timing of hydrolytic enzyme production (Hakkinen et al., 2014). Cellulase gene expression is adaptive, and in *T. reesei* it is regulated by the action of at least five transcriptional activators (XYR1, ACE2, ACE3, BgIR, and the HAP2/3/5 complex) and two repressors (ACE1, and the carbon catabolite repressor CRE1, Sternberg and Mandels, 1979; Aro et al., 2005; Stricker et al., 2007; Dashtban et al., 2011). However, how these physiological inducers control the activity of downstream transcription factors, or even what transcription factors are involved in cellulase and hemicellulase regulation, is not fully understood (Tani et al., 2014). Therefore, understanding how a filamentous fungus produces enzymes of industrial interest is a key step in obtaining improved strains for the production of enzymatic cocktails.

Here, we focused on XYR1 a major transcription factor involved in the regulation of hydrolytic enzymes (Stricker et al., 2006; Mach-Aigner et al., 2008) in *T. reesei*, by analyzing the influence of it absence on *T. reesei* gene expression. We used the Illumina RNA-seq technology to perform a comprehensive transcriptome analysis of *T. reesei* mutant strain (Δ*xyr1*) compared to *T. reesei* parental strain (QM9414), using two induction conditions (cellulose and sophorose) and a repressive condition (glucose) as sole carbon sources. Therefore, RNA-seq combined with bioinformatics provided an appropriate approach to study gene expression dynamics at a global scale during the developmental processes, allowing specific candidate genes to be highlighted for further functional analysis.

## Materials and methods

### Strains, media, and growth conditions

*T. reesei* strain QM9414 *(ATCC 26921)* and the Δ*xyr1* mutant strain (Stricker et al., 2006) were used in this study. The strains were maintained on MEX medium (malt extract 3% (w/v) and agar-agar 2% (w/v)) at 4°C. *T. reesei* QM9414 and Δ*xyr1* strains were grown on MEX medium at 28°C for 7–10 days until sporulation was complete. For gene expression assays, a spore suspension containing approximately 10^7^ cells mL^−1^ was inoculated into 200 mL of Mandels-Andreotti medium (Schmoll et al., 2009) containing 1% (w/v) cellulose (Avicel), 2% (w/v) glucose, or 1 mM sophorose, as the sole carbon source. The cultures were incubated on an orbital shaker (200 rpm) at 28°C for 24, 48, and 72 h for cellulose experiments; 24 and 48 h for glucose experiments; and 2, 4, and 6 h for sophorose experiments. The Δ*xyr1* strain was previously grown in 1% (W/V) glycerol for 24 h and then transferred to a medium containing cellulose for 8 and 24 h according to Stricker et al. (2006). All experiments were performed in three biological replicates. The resulting mycelia were collected by filtration, frozen in liquid nitrogen, and stored at −80° for RNA extraction.

### RNA extraction, library construction, and sequencing

Total RNA was extracted from mycelia of each sample using the TRIzol® RNA kit (Invitrogen Life Technologies, CA, USA), according to the manufacturer's instructions. RNA concentration was determined by spectrophotometric OD at 260/280, and the RNA integrity was verified by both the Agilent 2100 Bioanalyzer and gel electrophoresis in 1% agarose. The total RNA of the three biological replicates, cellulose (24, 48, and 72 h; 8 and 24 h for Δ*xyr1* strain), sophorose (2, 4, and 6 h), and glucose (24 and 48 h) of each strain were pooled, resulting in 18 samples used for library preparation (Figure S1), using the Illumina TruSeq RNA Sample Preparation Kit (Illumina) according to manufacturer's instructions. The pooled RNA samples were lyophilized and stored using the RNAstable® tube kit (Biomatrica) in order to maintain the RNAs integrity for sequencing. Sequencing libraries were prepared and sequenced by LGC Genomics GmbH (Berlin/Germany) on the Illumina Hiseq™ 2000 platform with paired-end 100 bp reads.

### RNA-seq data analysis

Illumina Hiseq™ 2000 was used to perform sequencing, resulting in 147 million and approximately 187 million 100 bp paired-end reads for parental (QM9414) and mutant (Δ*xyr1*) strains, respectively. Quality-filtered reads were mapped to the *Trichoderma reesei* 2.0 reference genome, available at JGI Genome Portal (http://genome.jgi-psf.org/Trire2/Trire2.home.html), using the TopHat2 v2.0.4 aligner (Kim et al., 2013), allowing two mismatches and only unique alignments. After alignment, Samtools version 0.1.18 (Li et al., 2009) was used to process the alignments files, which were visualized using the Integrative Genomics Viewer (Thorvaldsdottir et al., 2013). Htseq version 0.6.0 was used to count reads mapped to *T. reesei* transcripts. Genes were annotated using *Trichoderma reesei* 2.0 reference genome; and a local database provided by Prof. C. P. Kubicek (TU, Vienna) and the InterPro database (http://www.ebi.ac.uk/interpro/) (Jones et al., 2014; Mitchell et al., 2015). The R package DESeq2 version 1.6.3 (Love et al., 2014) was used to perform the differential expression analysis, using the raw number of reads mapped to each gene in each sample to perform statistical tests, based on the negative binomial distribution, which indicate whether a gene is differentially expressed in a condition relative to each other. Therefore, the DESeq2 package was utilized for normalization, using the median log deviation, and for the differential expression analysis, applying a log_2_ fold change ≥ 1 or ≤ −1 and an adjusted *p* ≤ 0.05 as thresholds. Thus, log_2_ fold change values ≥ 1 are up-regulated and log_2_ fold change ≤ −1 are down-regulated, case, present *p* ≤ 0.05.

### Functional enrichment

Differentially expressed transcripts were annotated using Blast2GO (Conesa et al., 2005). Functional enrichment analysis of differentially expressed genes based on Gene Ontology (GO) terms was performed using the BayGO algorithm (Vêncio et al., 2006). GO terms significantly enriched, i.e., *p* ≤ 0.05 were further analyzed, using GraphPad Prism v 5.00 Software.

### Network analysis

To reconstruct the regulatory network of Δ*xyr1*/QM9414 under the experimental conditions analyzed, differentially expressed genes (1184 in total) were analyzed using the Cytoscape 3.0.1 software (Shannon et al., 2003), following the same procedure reported by Dos Santos Castro et al. (2014).

### Phylogenetic analysis of transport-related proteins

A data set with 77 transport-related proteins sequences from *T. reesei* was selected for phylogenetic analysis. The amino acid sequences of transporters from *T. reesei* and other species (*Saccharomyces cerevisiae* YHR094C, YHR092C, YDR343C; *Neurospora crassa* NCU00821, NCU04963, NCU08114, NCU10021; *Aspergillus nidulans* AN3199, AN6831; *Aspergillus oryzae* AO090103000130, AO090038000233; *Aspergillus niger* XP_001397059.2; *Escherichia coli* YP006127731.1; *Metarhizium anisopliae* GQ167043.1, EFY97396.1, EFY94560.1; *Talaromyces marneffei* XP002152585.1; *Talaromyces stipitatus* XP002484658.1; *Candida albicans* 19.13383, *Ogataea angusta* ACA58225.1; *Beauveria bassiana* EJP62811.1; *Togninia minima* EOO00826.1; *Colletotrichum gloeosporioides* EQB50276.1 and *Fusarium fujikuroi* CCT62487.1) were obtained from JGI Genome Portal and other online databases (*Saccharomyces* Genome Database, *Neurospora* crassa Database, AspGD, GenBank, and *Candida* Genome Database). Sequences were aligned using MUSCLE and a phylogenetic tree was estimated by the maximum likelihood (ML) method using MEGA package version 6 (Tamura et al., 2013). A secreted lipase (ID 57204) from *T. reesei* was used as an outgroup.

### Real-time PCR (qRT-PCR) analysis

For quantitative real-time PCR (qRT-PCR) validation experiments, 20 genes were selected according to RNA-seq data (Table S1). For this analysis, 1 μg of RNA was treated with DNAseI (Fermentas) and reverse-transcribed to cDNA using the First Strand cDNA kit Maxima™ Synthesis (Thermo Scientific) according to manufacturer's instructions. The cDNA was diluted 1/50 and used for real-time PCR analysis in the Bio-Rad CFX96™ System, using SsoFastTMEvaGreen®Supermix (Bio-Rad) for signal detection in accordance with the manufacturer's instructions. The gene encoding actin (*act*) was used as endogenous control according to Steiger et al. (2010). The following amplification conditions were used: 95°C for 10 min followed by 39 cycles of 95°C for 10 s, 60°C for 30 s followed by a dissociation curve of 60°C to 95°C with an increment of 0.5°C for 10 s. Gene expression values were calculated according to the 2^−ΔΔCT^ method (Livak and Schmittgen, 2001) using the QM9414 strain grown on cellulose or sophorose as the reference sample. Primers used in the qRT-PCR experiments are described in Table S1. Data analysis was performed using GraphPad Prism v 5.0 Software.

### Statistical analysis and data records

All statistical analyses were performed using the R package (Team, 2011). RNA-Seq data from 18 libraries sequenced were deposited in the Gene Expression Omnibus (GEO) under accession number GSE66982.

## Results

### Analysis of RNA sequencing data

*T. reesei* QM9414 and the mutant strain Δ*xyr1* were cultivated in cellulose, sophorose, and glucose as unique carbon sources (Figure S1) and three biological replicates of each condition were submitted for RNA sequencing using Illumina HiseqTM 2000. Approximately 147 million of 100 bp paired-end reads were obtained for QM9414 and 187 million for the mutant strain Δ*xyr1*, with a concordant pair alignment rate of 89.8 and 89.2%, respectively. Regarding the number of nucleotides, the data obtained correspond to 38 GB for QM9414 and 45 GB for Δ*xyr1* (Tables S2, S3, respectively). The quality of data generated in sequencing was evaluated by the DESeq2 package and a high Pearson correlation was obtained when comparing the corresponding replicates of QM9414 (*R* ≥ 0.71) and Δ*xyr1* (*R* ≥ 0.67) for the three studied conditions (Figures S2, S3). A principal component analysis (PCA) (Figure S4) and box plot graphics (Figure S5) were performed to ensure the reliability of the sequencing data and demonstrate that the samples are comparable.

**Figure 1:**
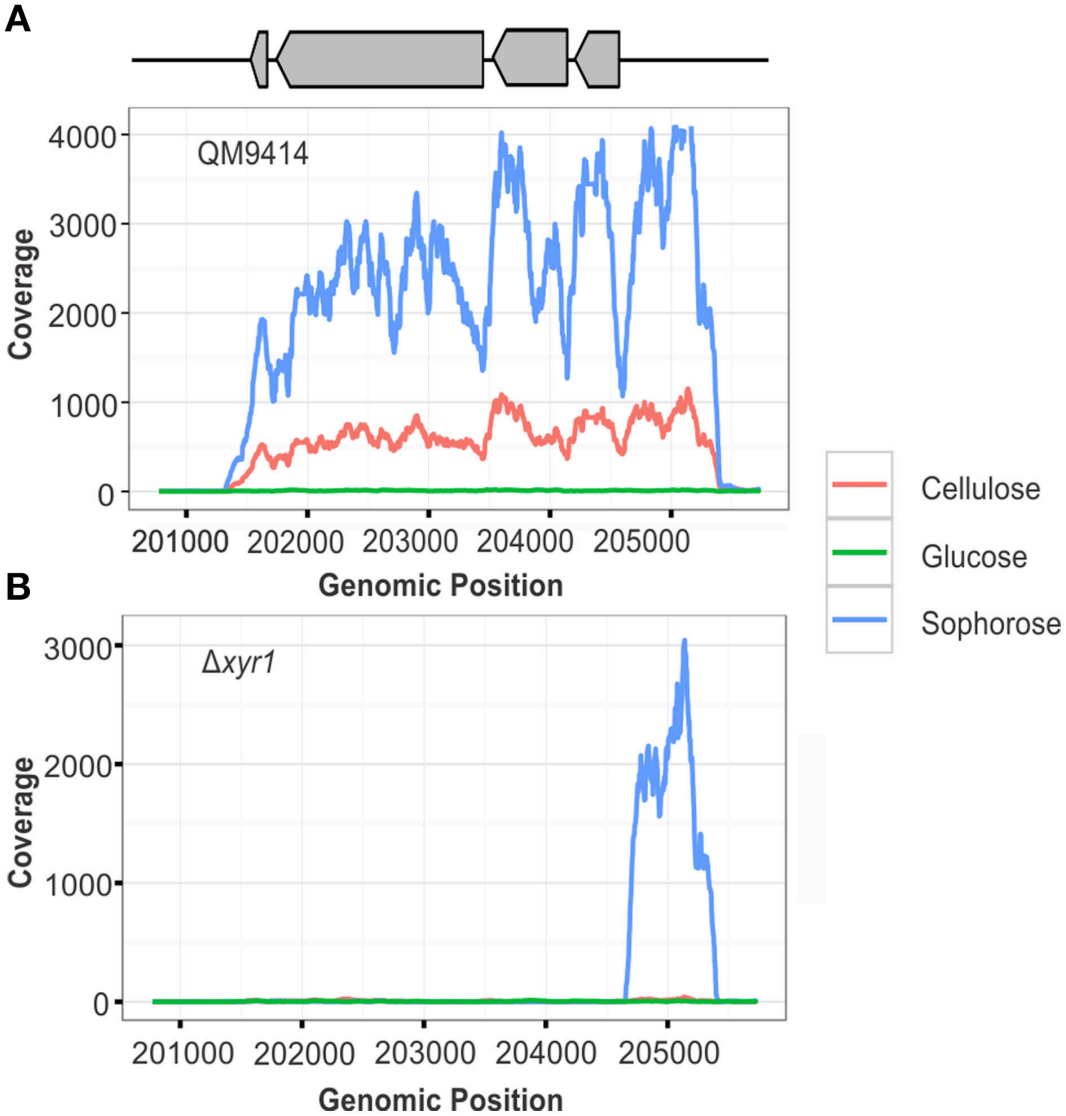
**RNA-seq coverage plots of the ***xyr1*** gene**. Number of sequence reads (x-axis) along the genomic region corresponding to the *xyr1* gene (Protein ID 122208, scaffold 11, genomic position 201774–204723).

To confirm the deletion of the gene *xyr1* in the mutant strain Δ*xyr1*, the files generated after the alignment of reads were visualized using IGV and the number of reads mapping to gene expression level of *xyr1* (ID 122208) was analyzed. It was possible to observe the absence of coverage in *xyr1* in the mutant strain for all conditions examined, confirming the presence of the mutation and eliminating any possibility of contamination during growth of the strains (Figure 1B). As expected, a large number of reads mapping to *xyr1* are observed in the QM9414 strain during growth in cellulose and sophorose. Regarding growth in glucose, coverage was observed in the parental strain, since the growth of *T. reesei* in readily metabolisable carbon sources does not require the presence of this transcription factor (Figure 1A).

To validate our RNA-Seq data, 20 genes modulated by XYR1 in the studied conditions were randomly chosen (Table S13). Among these genes, 6 were upregulated and 14 were downregulated in cellulose. Conversely, only one gene was upregulated in the Δ*xyr1* in sophorose. The log_2_ fold change of gene expression (Δ*xyr1*/QM9414) between the two comparisons obtained by RNA-seq and RT-qPCR demonstrated a significant Pearson correlation (*R*^2^ = 0.81), indicating the reliability of the RNA-seq analysis (Figure S7).

### Defining the transcriptional profile of XYR1 using RNA-seq

**Figure 2:**
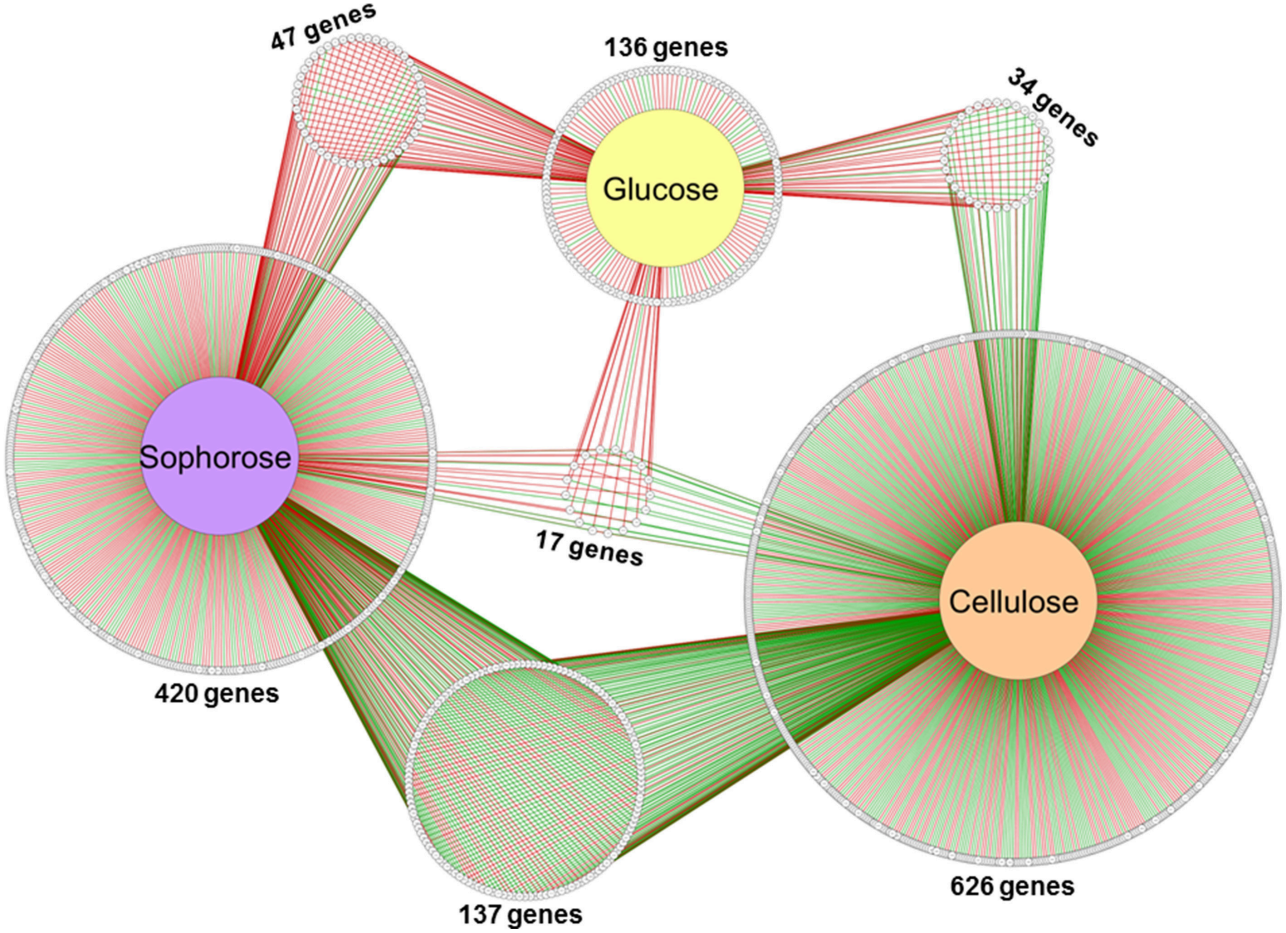
**Gene Regulatory Network (GRN) of 1184 differentially expressed genes in Δ***xyr1***/QM9414 in response to different carbon sources (cellulose, sophorose, and glucose)**. Genes are represented as nodes (shown as circles), and interactions are represented as edges (red lines: up-regulated interactions; and green lines: down-regulated interactions), connecting the nodes: 1674 interactions.

Of the 9129 genes present in the genome of *T. reesei*, 2359 were differentially expressed in the mutant Δ*xyr1* compared to QM9414 in the presence of cellulose, 2706 in sophorose, and 820 in glucose, adopting a *p* ≤ 0.05 as the threshold (Figure S6). Among these, a total of 877 were upregulated (*p* ≤ 0.05 and log_2_ fold change ≥1), with 322 in cellulose, 367 in sophorose, and 188 in glucose. Of the 796 genes that were downregulated (*p* ≤ 0.05 and log2 fold change ≤ −1), 492 were identified in cellulose, 255 in sophorose, and 49 in glucose (Figure S6). From these data, a regulatory network was built to identify genes that are specific of a determined growth condition (Figure 2). In this network, 626 genes were exclusively expressed during growth in cellulose, 420 exclusively in sophorose, and 136 exclusively in glucose, while only 17 genes were commonly expressed between the three conditions examined.

**Figure 3:**
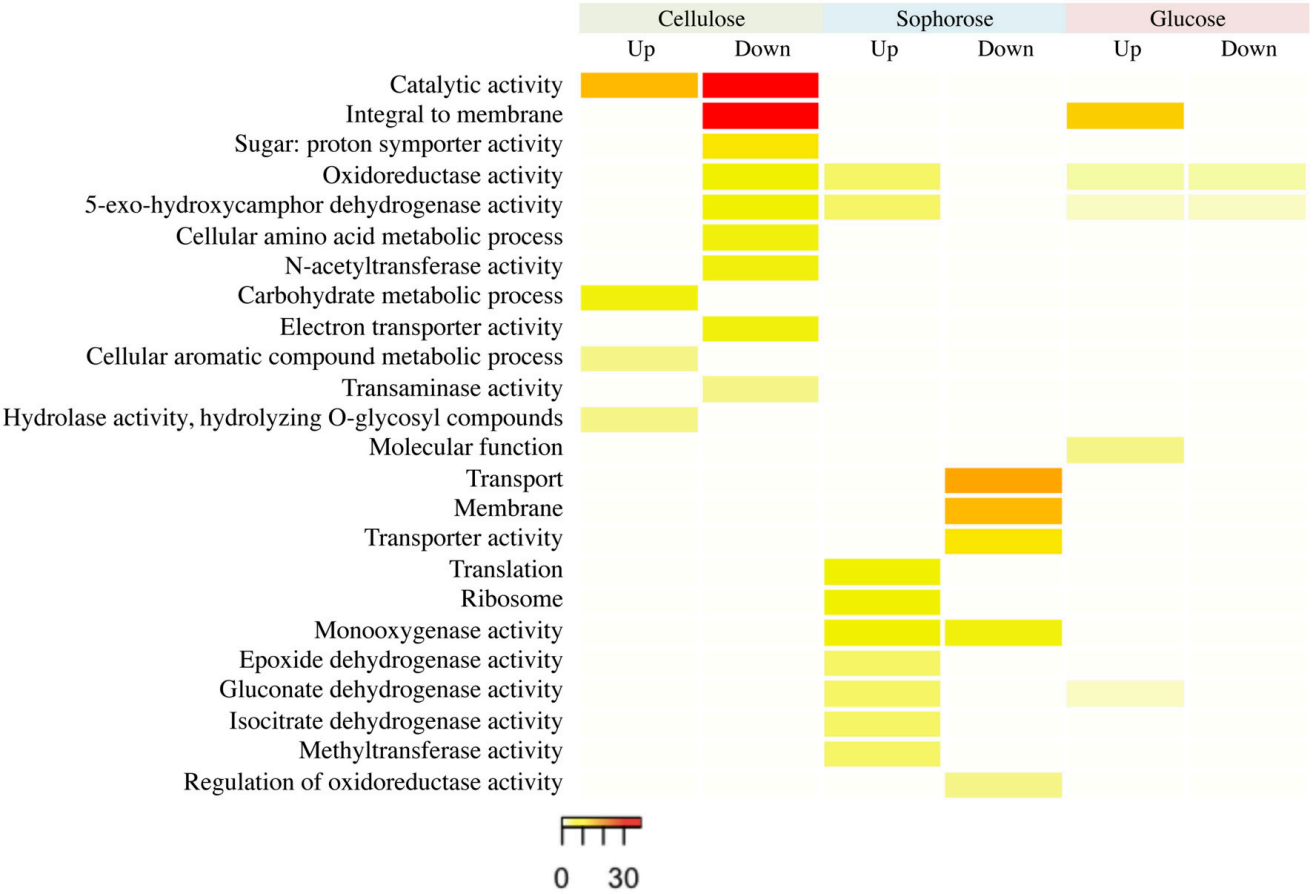
**GO enrichment analysis of up- and down-regulated genes of the Δ***xyr1*** strain compared to parental strain QM9414 grown in cellulose, sophorose, and glucose as sole carbon sources**. The enriched GO terms according to molecular function, cellular component, and biological process in *T. reesei*. Significantly enriched categories (*P* ≤ 0.05) are shown.

The functional categorization of the genes up- and downregulated in the mutant Δ*xyr1* compared to QM9414 was performed using the terms of Gene Ontology (GO) (Figure 3). The enrichment analysis showed that, in presence of cellulose, most of the upregulated genes are related to functions such as catalytic activity and carbohydrate metabolism. Among the downregulated genes in the same condition, the highlighted have catalytic activity function and are integral to the membrane. In presence of sophorose, the categories with the highest number of genes upregulated are translational, ribosomal, and those with monooxygenase activity, while downregulated genes are mainly related to transport and the membrane. The integral membrane and molecular function categories comprise the largest number of genes upregulated in glucose, while the categories of oxidoreductase activity and 5-exo-hydroxycamphor dehydrogenase activity were the only ones downregulated in this last condition (Figure 3).

### Major genes induced by XYR1 in cellulose, sophorose, and glucose

After an overview of the Δ*xyr1* transcriptome, we selected the top 15 genes that showed an induced expression by XYR1 transcription factor during growth in the different carbon sources analyzed (Table S4). In the inducing conditions with cellulose and sophorose, the majority of genes induced by the transcription factor XYR1 are related to proteins which act in the metabolism of carbohydrates, such as glycosyl hydrolases, carbohydrate esterases, and Carbohydrate Binding Modules (CBM).

In cellulose, the most induced gene by XYR1 is an α-xylosidase/α-glucosidase classified as family GH31 (ID 69944), which was at least 1260 times (log2 fold change = −10.3) less expressed in the mutant Δ*xyr1* when compared to the parental QM9414. Additionally, the xylanase enzyme (ID 120229) and the polysaccharide monooxygenase CEL61b (ID 120961), showed a 749- and 564-fold decrease in gene expression in Δ*xyr1*relative to QM9414 (log2 fold change = −9.55 and −9.14, respectively).

Similarly, another gene from the glycosyl hydrolases family, a β-xylosidase BXL1 (ID 121127), is the most XYR1 induced gene during cultivation in sophorose, achieving an expression level more than 209 times lower in the mutant Δ*xyr1* compared to QM9414 (log2 fold change = −7.71). Right after, are the carbohydrate esterase from the family 16 (ID 121418) and the α-Glucuronidase GLR1 (ID 72526), which were about 208 and 197 times less expressed in the mutant strain (log2 fold change = −7.70 and −7.62, respectively).

Still with regards to the inducing conditions, two transport-related genes were shown to be highly induced by XYR1 in cellulose and sophorose. They are the major facilitator superfamily (MFS) permeases ID 69957 (log2 fold change = −8.50) and ID 50894 (log2 fold change = −5.93), respectively, indicating that sugar transport can also be controlled by the transcription factor XYR1. The repressing condition glucose showed a heterogeneous population of genes induced by XYR1. The most repressed gene in the mutant Δ*xyr1* compared to QM9414 is the α-1,2-mannosidase from family GH92 (ID 60635), followed by an amidase (ID 109378) and a protein with unknown function (ID 108096), whose log2 fold changes are −2.89, −2.27, and −2.14, respectively.

### Role of XYR1 for the nutritional adaption of *T. reesei* to alternative carbon sources

**Figure 4:**
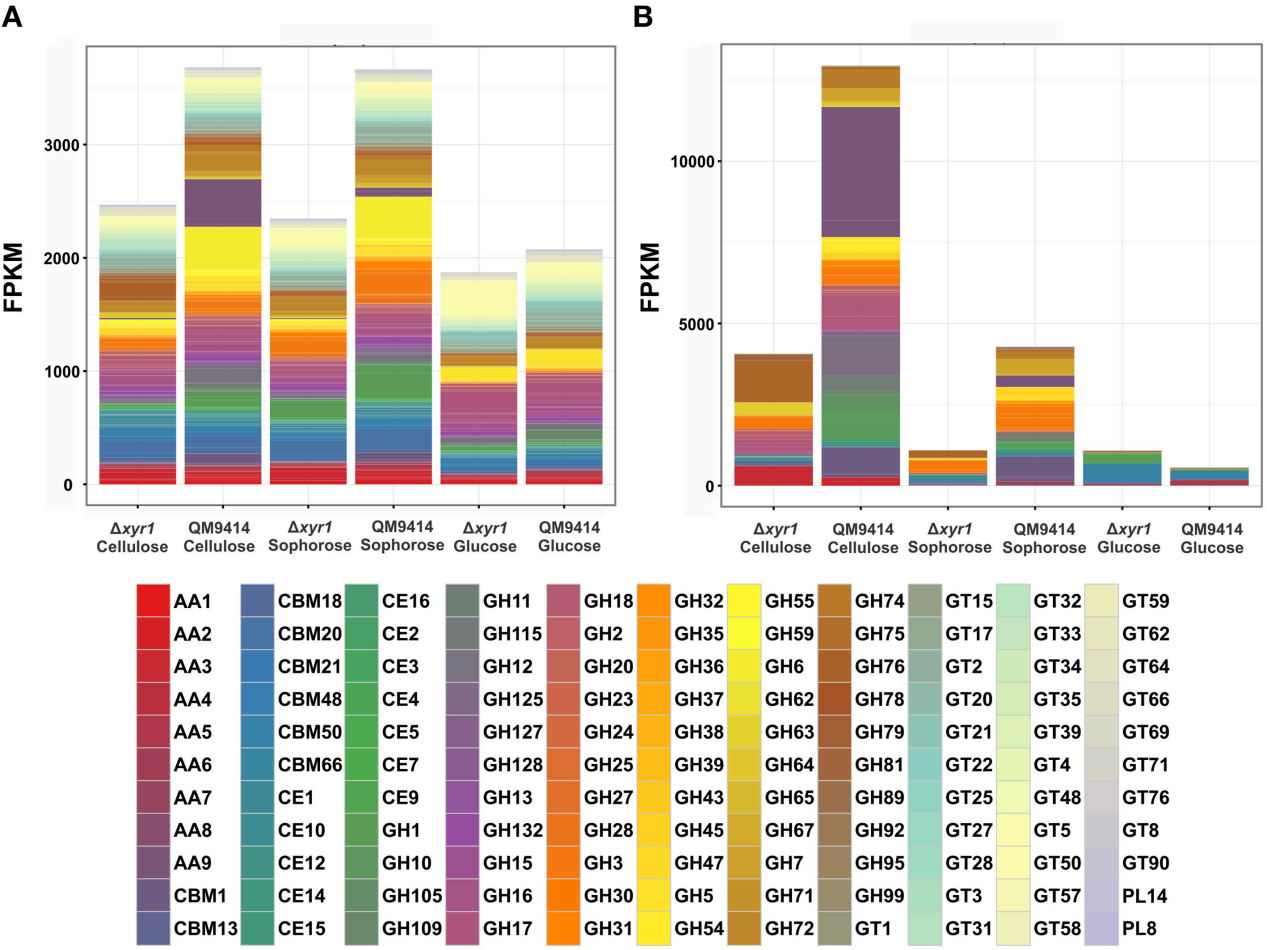
**Gene expression profiles of Carbohydrate Active enZymes (CAZy) of the Δ***xyr1*** strain compared to parental strain QM9414**. Fragments per kilobase of exon per million fragments mapped (FPKM) for each enzyme class cultured in presence of glucose, cellulose, and sophorose. **(A)** All CAZymes identified in this study; **(B)** Only the differentially expressed CAZymes of each carbon source were used in this analysis. AA, Auxiliary Activities—redox enzymes that act in conjunction with CAZymes; CBM, Carbohydrate-Binding Modules—adhesion to carbohydrates; CE, Carbohydrate Esterases—hydrolysis of carbohydrate esters; GH, Glycoside Hydrolases—hydrolysis and/or rearrangement of glycosidic bonds; GT, GlycosylTransferases—formation of glycosidic bonds; PL, Polysaccharide Lyases—non-hydrolytic cleavage of glycosidic bonds.

To access the role of XYR1 on CAZymes, the mean FPKM (fragments per kilobase of exon per million fragments mapped) for all the genes within a single GH family were calculated. The total of all the FPKM means for each GH family in the inducing carbon sources cellulose and sophorose, and in the repressive source glucose were utilized to demonstrate the global transcriptional gene response (Figure 4). As expected, during growth in the presence of glucose the entire transcription of GH encoding genes was low, whereas growth in the presence of cellulose or sophorose resulted in a dramatic induction of a wide array of GH families, reflecting the transcriptional induction of the CAZymes. In the QM9414 parental strain in the presence of cellulose, the expression of the members of GH families 61(AA9), 12, and 1 were overexpressed (Figure 4B). On the contrary, in the Δ*xyr1* mutant strain we observed the induction of the expression of the members of GH families 76, 64, and 3, as well as the members of the auxiliary activity enzymes of the family AA3. Similarly, in presence of sophorose in both QM9414 and Δ*xyr1* strains, the high expression of cellobiohydrolase members from the GH3 and carbohydrate esterases CE 1 was observed. Furthermore, in the parental strain we demonstrated the induction of expression of members of the AA7 family, GH 6, and GH 1 families.

In the presence of a repressive carbon source, the overall transcriptional response was slightly higher in the Δ*xyr1* mutant strain and the expression of the members of the CBM50 (Carbohydrate Binding Modules), CBM 13, and AA5 families were prevalent (Figures 4A,B).

We were also interested to know if the regulation of CAZymes by XYR1 is direct or indirect. A deeper analysis of Table S9 shows the CAZy genes that are directly regulated by XYR1. Interestingly, two members of redox enzyme families AA9 and AA7 were shown to be directly downregulated and upregulated in the Δ*xyr1* mutant strain in the presence of cellulose and sophorose, respectively. Likewise, in the presence of cellulose, a decreased expression of members of GH 17, 27, 3, 31, 67, and 64 was observed in Δ*xyr1*. Similarly, in sophorose, a decrease in the expression of GH 27, 3, 67, and PL8 polysaccharide lyase family members was also seen for the mutant strain. These results indicate that the expression profiles of GH members in the Δ*xyr1* are closely similar when grown in the presence of cellulose and sophorose. This resemblance is probably due to the sophorose released by cellulose depolymerization during deconstruction of lignocellulosic material. In Tables S6–S8 we observed the genes that are exclusively regulated in the presence cellulose, sophorose, and glucose, respectively. Among these, 16 genes were upregulated in presence of cellulose, 8 in sophorose, and 5 in glucose in the Δ*xyr1*. On the other hand, a higher downregulation of the CAZy genes was seen in the presence of cellulose (36 genes), and sophorose (16 genes). Additional information about all CAZys identified in this study is described in the Table S5. Taken together the results showed that although the profile of cellulase expression seems to be similar in the presence of cellulose and sophorose, the general regulatory mechanism employed by XYR1 to control the expression of specific genes was shown to be specific to the carbon source.

To achieve nutrition, is important that the fungus has efficient nutrient transporters. In the *T. reesei* genome, approximately 5% (459 genes) of the genes encode proteins with transport function. Among these genes, 16%, or 77 genes, encoding transporter activity were shown to have their expression modulated in the Δ*xyr1* mutant strain compared to QM9414 (Table S12). The modulation of transporter expression was shown to be carbon source-dependent, with a total of 35 upregulated genes and 42 downregulated genes in the mutant strain. In presence of cellulose, 13 genes were upregulated and 29 downregulated for a total of 42 genes with changed expression profiles. When cultured in the presence of sophorose, the Δ*xyr1* mutant strain showed an upregulation and downregulation of the 16 and eight transporter genes, respectively. The profile of transporter gene expressions in the presence of glucose was less evident than in cellulose and sophorose. In the repressive condition, 11 transporter genes had their expression modulated, with six and five genes upregulated and downregulated, respectively.

**Figure 5:**
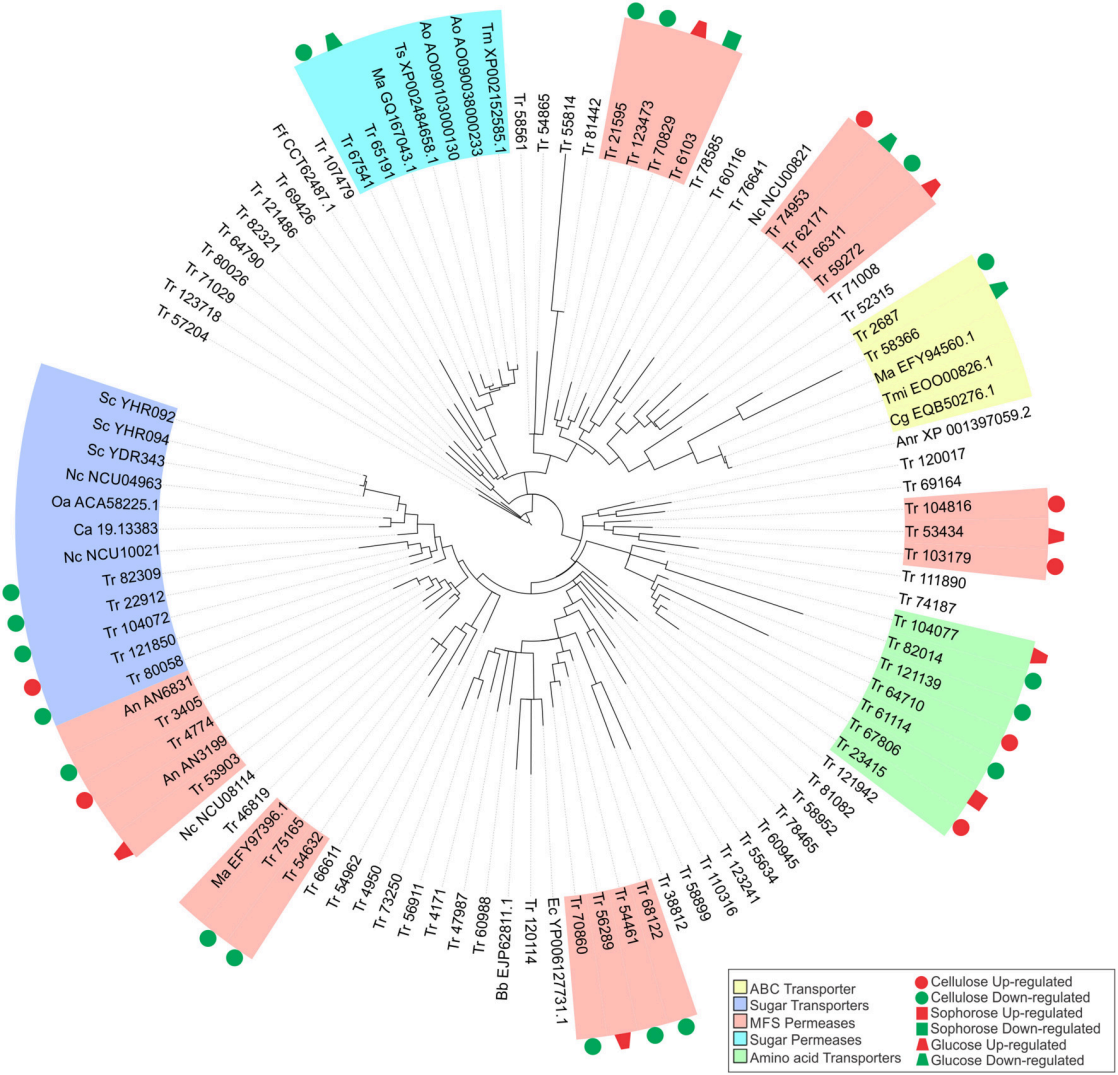
**Maximum-likelihood phylogenetic tree of transporters genes identified exclusively in the conditions studied**. The radial tree was generated using iTOL (Letunic and Bork, 2007, 2011).

Five transporter families as well as the ABC transporter, sugar transporter, MFS permeases, sugar permeases, and amino acid transporters were preferentially modulated by XYR1 in the presence of cellulose, sophorose, and glucose (Figure 5). Almost half of these genes encode proteins that belong to the MFS permeases. In the presence cellulose, we observed the expression of 21 MFS genes with 16 genes being downregulated and five upregulated in the Δ*xyr1* strain. Eight genes (six upregulated and two downregulated) in sophorose and four genes (two upregulated and two downregulated) in glucose also belong to the group of MFS permeases (Table S12).

The gene that was most repressed by XYR1 in the presence of cellulose encodes a MFS permease (ID 103179), which was had a 24 times (log_2_ fold change = 4.6) higher expression in the mutant Δ*xyr1* compared to the parental strain, QM9414. Conversely, in sophorose and glucose, the two genes most repressed by XYR1 were the copper transporter ctr (ID 52315) (log_2_ fold change = 7.74, 213 times) and the ZIP Zinc transporter (ID 47987) (log_2_ fold change = 1.85, 4 times), respectively. Three genes encoding an urea transporter (ID 56911) (log_2_ fold change = −7.35, 163 times), an AAA+-type ATPase (ID 58366) (log_2_ fold change = −5.67, 51 times), and a putative MFS multidrug transporter (ID 6103) (log_2_ fold change = −1.91, 3.8 times) were the most downregulated genes in the Δ*xyr1* mutant strain in cellulose, sophorose, and glucose compared to QM9414 parental strain. The lactose permease ID 3405 also showed a strong downregulation in the mutant strain in presence of cellulose. Furthermore, amino acid, calcium, copper, and iron are the main transporter families with modulated expression in the mutant strain in the three carbon sources. Among the transporters, only the MFS permeases ID 55077 and ID 80058, and the amino acid transporter ID 123718 were directly regulated by XYR1. These transporters were only modulated in the presence of cellulose.

In order to better understand the function of each target transporter of XYR1-mediated modulation in the presence of cellulose, sophorose, and glucose, we performed a phylogenetic analysis using the amino acid sequences of the transporters upregulated and downregulated in Δ*xyr1* compared to QM9414 (Figure 5). The transport-related proteins of different species were also included in the analysis (See Materials and Methods Section). A secreted lipase (ID 57204) of *T. reesei* was used as an outgroup. Our results showed that the two largest clusters were formed by sugar transporters and MFS permeases. In the same way, three other groups including sugar permeases, ABC transporters, and amino acid transporters were also obtained (Figure 5). The group related to the ABC transporters is comprises of two *T. reesei* ABC transporters and three from *Colletotrichum gloeosporioides, Togninia minima*, and *M. anisopliae*. These two *T. reesei* ABC transporters were downregulated in the presence cellulose and glucose in the mutant strain. The cluster of amino acid transporters includes seven proteins of *T. reesei* with two proteins being upregulated on cellulose and one protein being upregulated in both sophorose and glucose in Δ*xyr1*. Moreover, in this cluster we still have three downregulated proteins in the presence of cellulose.

Five MFS permeases and five sugar transporters from *T. reesei* are similar to 10 sugar transporters from other species, suggesting that these *T. reesei* transporters are involved in the uptake of sugars into the cell. Another cluster was formed by two *T. reesei* sugar permeases (ID 65191 and ID 67541), one of which has previously been described as a MFS maltose permease, and these are similar to another five sugar permeases belonging to *Talaromyces stipitatus, Aspergillus oryzae*, and *Metarhizium anisopliae*. This *T. reesei* transporter and other maltose and sugar transporters from *Aspergillus oryzae, Talaromyces marneffei, Metarhizium anisopliae*, and *Talaromyces stipitatus*, are possibly involved in the transport of disaccharides, such as cellobiose or sophorose, which are produced as a result of the degradation of the cellulose polymer by the cellulolytic complex.

### Global regulation by the Zn2Cys6 transcription regulator XYR1 in *T. reesei* cellulase expression

**Figure 6:**
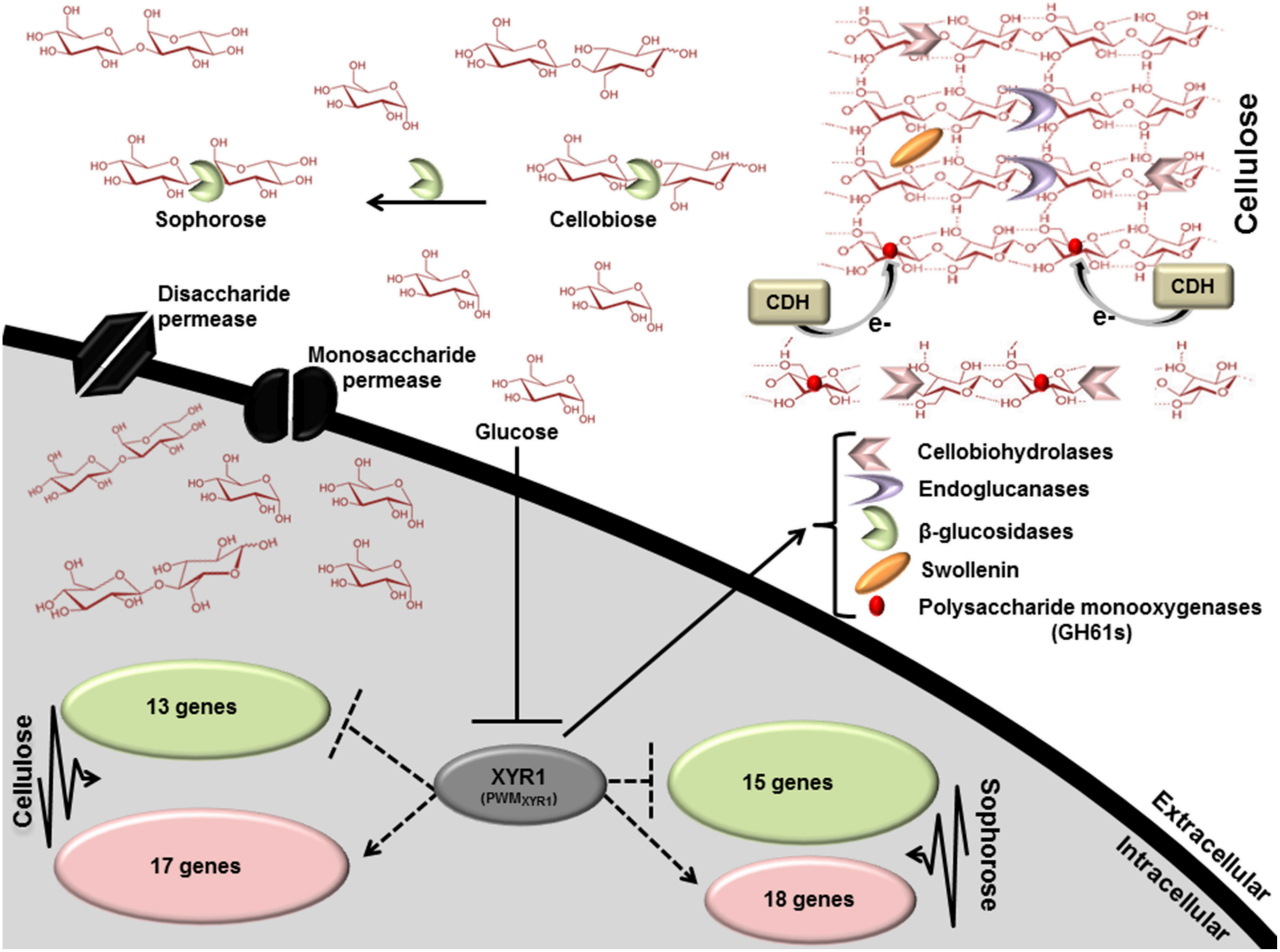
**Scheme involving the direct regulation by the Zn2Cys6 transcription regulator XYR1**. This figure shows the processes that are expected to be involved in *xyr1* regulation of lignocelluosic enzymes in *T. reesei*. Endoglucanases (EGs) hydrolyze cellulose bonds internally, while cellobiohydrolases cleave cellobiose units from the ends of the polysaccharide chains. The released cellobiose units (disaccharides) are subsequently hydrolyzed by β-glucosidases, releasing glucose, the main carbon source readily metabolisable by *T. reesei*. Expansin proteins (Swollenin) rapidly induce extension of plant cell walls by weakening the noncovalent interactions that help to maintain their integrity. Cellobiose dehydrogenase (CDH) is a potential electron donor for polysaccharide monooxygenases (PMOs). EGs and PMOs internally cleave cellulose chains releasing chain ends that are targeted by cellobiohydrolases (CBHs). Dotted arrows indicate potential areas of research for enhancement of enzymes secretion in *T. reesei*: XYR1 activates or represses (directly) the transcription of genes in cellulose and sophorose. PWM_*XYR1*_ defined in Silva-Rocha et al. (2014).

To better understand the general mechanism of cellulase gene expression in *T. reesei*, we constructed a global scheme to clarify the exercised control profile by transcription factor XYR1. In the Figure 6, we demonstrated the processes that are expected to be involved in the regulation of lignocellulosic enzymes in *T. reesei*. This fungus has three distinct classes of enzymes, endoglucanases, cellobiohydrolases, and β-glucosidases that synergistically breaks down the cellulose polymer. Endoglucanases (EGs) act by cleaving internal β-glycosidic bonds in the cellulose chain, thereby making chain ends accessible to cellobiohydrolase. The end product, cellobiose, is further broken down to units by β-glucosidase, releasing glucose, the main carbon source that is readily metabolisable by *T. reesei*. Our results demonstrated that different glycosyl hydrolases had their expression modulated in the presence of cellulose, sophorose, or glucose by XYR1. Sixteen CAZyme genes were shown to be directly regulated by XYR1 (Table S9). We demonstrated that the Δ*xyr1* mutant strain has a similar downregulated gene profile in the presence of cellulose and sophorose. In these conditions, genes encoding GH 31, GH 61, GH 5, GH 3, CE 15, CE 16, and CBM1 were the most commonly downregulated genes in the mutant strain, suggesting a functional role of XYR1 in the regulation of these genes. In addition, the main CAZyme genes downregulated in cellulose belong to the GH 10, GH 5, GH 11, GH 12, and GH62 families, whereas in sophorose, the major downregulated families are GH 67, GH 109, GH 7, and GH 30.

The general expression profile of transcription factors was highly modulated in the mutant strain (Table S10). However, none of the transcription factors known to regulate the expression of cellulase genes in *T. reesei* had their expression altered. Thirty-one transcription factors were modulated by XYR1 in presence of cellulose, 17 in sophorose, and seven in the presence of glucose. The most upregulated transcription factor in the mutant Δ*xyr1* in cellulose (ID 122271, log_2_ fold change = 3.49, 11 times) is a homolog of the Zn2Cys6 transcriptional regulator of *C. albicans* Fcr1, which has been described to be involved with ABC transporter expression regulation. A homolog to the *myb* gene (ID 4124), previously described as a regulator of nitrogen metabolism in *Aspergillus nidulans*, was 9.5-fold less expressed in the mutant strain compared to the parental QM9414. Curiously, another *myb* member family was one of the most upregulated transcription factors in cellulose (ID 1941, log_2_ fold change = 2.52, 5 times). This result suggests a refined dual mechanism that controls the expression of cellulase genes through the modulation of transcription factors.

The majority of existing knowledge related to monosaccharide uptake in fungi originates from studies in yeast *S. cerevisiae* models. This yeast is able to transport and metabolize glucose, fructose, mannose, and galactose. Transport of these simple sugars is mediated only through facilitated diffusion by the majority of the transporters from the Hxt family belonging to the sugar porter (SP) family, which is the largest subfamily of the MFS (Leandro et al., 2009). In this regard, two transporter genes belonging to the MFS family (ID 69957—cellulose and ID 50894—sophorose) were drastically downregulated in the mutant strain (Table S4). These genes are 361- and 61-fold more expressed in the parental strain QM9414, respectively. These findings suggest that MFS members have an important role in cellulase gene regulation in induction by both cellulose and sophorose, are involved in transporting sugars (disaccharides or monosaccharides) into the cell, and can be regulated by XYR1. Briefly, our global scheme (Figure 6) suggests that XYR1 indirectly regulates most of the genes in *T. reesei*, including those involved in cellulase expression. Overall, only 30 genes were directly regulated by XYR1 in presence of cellulose and 33 in sophorose, exclusively (Table S11). A functional XYR1 product may modulate the expression of different transcription factors that in turn regulate the expression of all necessary components involved with cellulase expression. This refined repertoire involves, as demonstrated by our results, the expression of specific glycosyl hydrolase family members, transcription factors, and transporters, with their expressions regulated according to carbon source.

## Discussion

We aimed to achieve a comprehensive understanding of how gene expression is regulated by XYR1 in *T. reesei* in response to different carbon sources by RNA-seq and bioinformatics analysis. By comparing the growth in cellulose and sophorose, we identified new features in cellulose degradation in *T. reesei* and showed a remarkable similarity between their transcriptomic profiles.

The expression of *xyr1* varies according to the analyzed condition. In cellulose and sophorose, the role of the XYR1 in the induction of cellulolytic and hemicellulolytic enzymes is already well-established (Stricker et al., 2006), justifying its high level of gene expression in the parental strain QM9414. Conversely, we observed that in the presence of glucose, the expression of *xyr1* was minimal (Figure 1), which may be related to the intervention of another transcription factor also involved in the regulation of cellulase expression in *T. reesei*, the carbon catabolic repressor CRE1. In 2014, Antoniêto et al. (2014) observed that in presence of glucose, the CRE1 transcription factor is triggered and represses *xyr1* expression by approximately 36-fold, thus explaining the lower *xyr1* expression level in the presence of glucose.

Although, others transcriptional factors such as ACE2, ACE3, HAP2/3/5 complex (positive regulators), ACE1, and CRE1 (negative regulators) are involved in *T. reesei* cellulase gene expression (Kubicek et al., 2009; Seiboth et al., 2012; Hakkinen et al., 2014), numerous studies report that XYR1 is the main factor between positive cellulase transcriptional activators, because its knock-out not only suppresses cellulase but also xylanase and β-mannanase formation (Mach-Aigner et al., 2008; Seiboth et al., 2012; Hakkinen et al., 2014; Tani et al., 2014). Stricker et al. (2008) showed that deletion of *xyr1* eliminates the induction of cellulases in the presence of both cellulose and sophorose, supporting its essential role in induction. In addition, a C-terminal truncation by 140 amino acids detected in the transcription factor XYR1 in the strain QM9136 (derived from QM6a) made this *T. reesei* strain unable to produce cellulase (Lichius et al., 2015). In *Aspergillus niger*, an XYR1 orthologue, XlnR, regulated the transcription of more than 20 genes encoding hemicellulases and cellulases (Stricker et al., 2008). de Souza et al. (2013) showed that different gene sets of CAZy enzymes and sugar transporters were shown to be individually or dually regulated by transcription factors XlnR and AraR, with XlnR appearing to be the major regulator of complex polysaccharides in *A. niger*. However, despite being highly conserved among filamentous fungi, XYR1 (XlnR/Xlr1) may exert different functions depending on the species (Klaubauf et al., 2014). In the filamentous fungus *Neurospora crassa*, the deletion of *xlr-1* (orthologue to XYR1 and XlnR) abolished its growth on xylan and xylose, but growth on cellulose and cellulolytic activity were only slightly affected (Sun et al., 2012). Unlike our observations in *T. reesei*, the overexpression of Mtxyr1 (XYR1 homolog) in *Myceliophthora thermophila* increased the xylanolytic activity in glucose and corncob; however, filter paper activity (FPA) and endoglucanase (EG) showed no significant difference compared to the parental strain (Wang et al., 2015). Furthermore, Li et al. (2015) also observed that in *Penicillium oxalicum* the lack of XlnR weakly reduced the transcript levels of some cellulases, but not at the same intensity as that found in *T. reesei*. Finally, in the plant pathogen *Magnaporthe oryzaeis*, no difference in the cellulolytic and xylanolytic profiles was observed when comparing the mutant strain Δ*xlr1* with its corresponding parental strain (Battaglia et al., 2013). Taken together, these data suggest that *T. reesei* specifically controls cellulase and xylanases gene expression by the transcription factor XYR1.

Interestingly, our data revealed that the main transcription factors that regulate the expression of cellulases in *T. reesei* showed no changes in expression in the mutant strain relative to QM9414 parental strain. Among them, only *ace3* (ID 77513) had a slight decreased expression in cellulose (fold change = −0.91, 1.88 times) (data not shown). Hakkinen et al. (2014) demonstrated that deletion of *ace3* in *T. reesei* significantly reduced xylanase activity and expression of xylan-degrading enzyme genes. Studies have shown that XYR1 deletion abolishes the expression of xylanase genes (Seiboth et al., 2012; Tani et al., 2014). As such, our results highlight a possible regulatory role of XYR1 on ACE3 in the expression of xylanase genes. This result was in concordance with a previous study described by Herold et al. (2013), which revealed that the expression of all xylanases, except *xyn3*, is induced by D-xylose and is XYR1-dependent.

Curiously, we demonstrated for the Δ*xyr1* mutant strain that *xyn3* was the second most downregulated gene (fold change = −9.55, 750 times; Table S4) in cellulose, indicating that its expression might be also regulated by XYR1 in this carbon source. According to Silva-Rocha et al. (2014), there is one binding site to XYR1 in the *xyn3* promoter region. However, we showed that *xyn3* is not directly regulated by XYR1, indicating a possible interplay in the regulation of xylanase gene expression involving other genes in *T. reesei*, and that *xyn3* expression of might be regulated by a XYR1-independent pathway. To address this, we performed an *in silico* analysis to identify cis-regulatory elements for XYR1 in the regulatory region of *ace3*. Our analysis showed that in the promoter region of *ace3* there are 2 binding sites for XYR1 (data not shown). This result evidences the regulation of xylanase genes under the control of an interplay between *xyr1* and *ace3*. Our results are in accordance with Sun et al. (2012) who showed that the regulation of genes encoding xylanolytic enzymes in *Neurospora crassa* involves several regulatory groups, where the XLR-1 (xylanase regulator) may work alone or in combination with other unknown regulators. Additionally, an XLR-1 independent group of genes was also suggested to exist.

Another xylanase gene downregulated in the mutant strain during growth in cellulose was a GH11 member *xyn2* (ID 123818). The expression of this gene may be induced by xylobiose, xylan, cellulose, and sophorose (Zeilinger et al., 1996). Our data show that it was expressed 384-fold lesser in the mutant strain than in the wild type, when growth in cellulose was not directly regulated by XYR1. Würleitner et al. (2003) demonstrated that in *T. reesei*, the regulation of *xyn2* expression is based on the interplay of Hap2/3/5, Ace2, and the AGAA-box binding repressor. Similarly, for *ace3*, our *in silico* analysis showed that there are 2 binding sites for XYR1 in the promoter region of Hap2 (data not shown). This result suggests that XYR1 might indirectly control the expression of *xyn2* by regulating the expression of components of the Hap2/3/5 complex, which is involved in xylanase gene expression.

Regarding the CAZy genes under direct XYR1 regulation, the most downregulated gene in the presence of cellulose encodes a GH61 polysaccharide monooxygenase protein CEL61a (AA9, Auxiliary family activity 9), which is also downregulated in sophorose (fold change = −8.30, 315 times and −5.97, 62.5 times, respectively). This enzyme has been shown to enhance lignocellulose degradation by an oxidative mechanism (Langston et al., 2011; Häkkinen et al., 2012). Our data corroborates the hypothesis of Quinlan et al. (2011) that GH61 glycoside hydrolases, reclassified as AA9, may act directly on cellulose to make it more accessible to traditional cellulases. Therefore, we suggest that this enzyme might also be involved in the metabolism of carbon sources less complex than cellulose (such as sophorose), becoming an important target of XYR1 responsible for initiating the degradation of available cellulose and equally responsible for oxidative degradation of these substrates.

Our results also show that cellulase induction involves a range of transcription factors that can be regulated by XYR1, mostly in an indirect manner. Furthermore, we demonstrated that the control of cellulase expression mediated by XYR1 does not necessarily occur by regulation of the main transcription factors involved with cellulase synthesis. Of the 55 transcription factors genes modulated by XYR1 in the studied conditions (Table S10), only 5 were directly regulated by XYR1 and none were previously characterized in *T. reesei*. In the presence of cellulose, the most upregulated transcription factor (ID 122271, fold change = 3.49, 11 times) is a homolog to *C. albicans fluconazole resistance 1 protein* (Fcr1p), a member of the family of zinc cluster proteins characterized by a highly conserved Zn(II)2Cys6 zinc finger motif within the N-terminal DNA binding domain (Talibi and Raymond, 1999). This transcription factor has been described to be responsible for the regulation of the expression of a large family of membrane transporters (ABC transporters; Dexter et al., 1994; Shen et al., 2007). The most repressed (ID 4124) and the third-most upregulated gene of transcription factors (ID 1941) in the presence of cellulose in the mutant strain belonged to the *Myb* family. These proteins are involved in several cellular processes in eukaryotes, including cell proliferation, apoptosis, differentiation, metabolism, and stress responses (Dubos et al., 2010; Ravaglia et al., 2013). Transcription factors (TFs) of the Myb family are found in all kingdoms of life (Stober-Grässer et al., 1992; Wieser and Adams, 1995; Oh and Reddy, 1999; Rubio et al., 2001; Du et al., 2009; Meneses et al., 2010) and have been described as regulators of nitrogen metabolism in *A. nidulans* and *Fusarium graminearum* (Arratia-Quijada et al., 2012; Kim et al., 2014). Additionally, Zhang et al. (2012) showed in *Phytophthora sojae* that an Myb transcription factor is required for zoospore development. Stricker et al. (2006) showed that Δ*xyr1* has similar growth rates and neither conidiospore formation nor germination times differed between the mutant and the parental strain. As a result, due to *myb* downregulation in the Δ*xyr1* mutant strain further studies will be performed to understand the role of Myb transcription factors in this fungus.

We demonstrated that 4 genes encoding ABC transporters are differentially expressed in the presence of cellulose, sophorose, and glucose, and are mainly downregulated under these conditions. Several studies have shown that the ABC transporter superfamily is involved with the intracellular transport of metabolites across bacterial membranes (Goffeau et al., 2004; Davidson et al., 2008). Moreover, in fungi, these transporters are involved with mycoparasitic interaction and antifungal resistance (Ruocco et al., 2009; Sa-Correia et al., 2009; Morschhäuser, 2010; Paul et al., 2013; Karlsson et al., 2015). Schlosser et al. (1999) described in *Streptomyces reticuli* an inducible ABC transport system of uptake specific for cellobiose and cellotriose. Similarly, Elferink et al. (2001) showed in *Sulfolobus solfataricus* (an extreme thermoacidophilic archaeon), that sugar transport is mediated by two families of protein binding dependent ABC transporters, which may transport arabinose, fructose, xylose, glucose, galactose, cellobiose, maltose, and trehalose. Similarly, Koning et al. (2001) showed that cellobiose uptake in the hyperthermophilic archaeon *Pyrococcus furiosus* is mediated by an inducible, high-affinity ABC transporter. Recently, Watanabe et al. (2015) related the functional characterization of β-xylosidase and the xyloside ABC transporter in a soil bacterium *Corynebacterium glutamicum*. The findings described by Watanabe and co-workers suggest some inherent ability of *C. glutamicum* to take up xylooligosaccharides, an ability that is enhanced by in the presence of a functional xyloside ABC transporter. However, in *T. reesei*, the function of the transporters belonging to the ABC family remains unclear. So, the downregulation of the ABC transporter family members in the Δ*xyr1* mutant strain suggests an important role for these transporters in cellulase gene expression under the control of XYR1, reinforcing the requirement of a functional and structural characterization of these transporters in *T. reesei*.

In addition to ABC transporters, two other classes of transporters related to amino acids and MFS transporters were highly modulated in the Δ*xyr1* mutant strain in the presence of cellulose, sophorose, and glucose. The MFS proteins represent a large family of secondary transporters carrying small solutes (Pao et al., 1998; Yan, 2015). At the moment, 74 families of MFS transporters have been classified, wherein each one transports a specific substrate as simple monosaccharides, oligosaccharides, amino acids, peptides, vitamins, enzyme cofactors, and others (Reddy et al., 2012). In yeast, transporters belonging to the MFS have been found to play important roles in sugar uptake (Bisson et al., 1993; Weusthuis et al., 1994; Fan et al., 2002). Fekete et al. (2012) identified a MFS lactose transporter important for lactose uptake and growth on lactose in *A. nidulans*. Additionally, Colabardini et al. (2014) identified the *A. nidulans xtrD* (xylose transporter) gene encoding a transporter from the MFS. This transporter is expressed in the presence of xylose in an XlnR-dependent manner, and is also able to transport xylose, glucose, galactose, and mannose, indicating that this transporter accepts multiple sugars as a substrate.

Porciuncula Jde et al. (2013) showed that the MFS transporters ID 3405, ID 79202, and ID 77517 are highly expressed during growth of *T. reesei* in cellulose and lactose. In this study, we demonstrated that the MFS transporter ID 3405 was downregulated (78-fold) in the Δ*xyr1* mutant strain grown in cellulose. Recently, our group also revealed that this transporter is strongly repressed by CRE1 when a metabolizable carbon source is present in the medium (Antoniêto et al., 2014). Moreover, Zhang et al. (2013) showed that this protein (named Ctr1) plays a key role in the cellulolytic signaling process, acting as sophorose transporter. Conversely, Ivanova et al. (2013) indicated that this gene is also upregulated in the presence of lactose, probably acting as lactose permease. The lack of specificity of these transporters could be explained by the similar structure of different sugars such as lactose/sophorose and beyond that, some transporters can act as both transporters and nutrient sensors. In presence of cellulose, another MFS permease (ID 69957) was specifically downregulated in the Δ*xyr1* strain. This transporter has a high similarity to a putative maltose permease of the human pathogenic fungus *Talaromyces marneffeii* and may be involved in the transport of disaccharides (Boyce and Andrianopoulos, 2013). On the other hand, the gene encoding an MFS hexose transporter (ID 46819), which was downregulated in the presence of cellulose in the Δ*xyr1* mutant is homologous to a putative cellodextrin transporter-like protein CLP1 of *Neurospora crassa*, which is involved in cellulase induction. Functionally, CLP1 cannot transport cellodextrin, but it can repress the expression of the cellodextrin transporter, in turn inhibiting cellodextrin uptake in *N. crassa* (Cai et al., 2015). Together our results suggest that XYR1 may regulate the expression of different transporters in *T. reesei* and these transporters have a promiscuous role in signaling pathways involved in cellulase induction.

Finally, our results demonstrated that genes related to amino acid transporters were mainly downregulated in the presence of cellulose and sophorose. Moreover, only one amino acid transporter was upregulated in the three tested carbon sources. Ivanova et al. (2013) showed that genes encoding proteins involved in amino acids metabolism were enriched in lactose-grown cultures. In addition, Antoniêto et al. (2014) showed that in the Δ*cre1* mutant strain the expression of amino acid transporters was induced in the presence of cellulose. In addition, during growth in sophorose, expression of the amino acid transporter in the Δ*cre1* mutant strain was also induced, indicating that CRE1 acts to inhibit these genes (Antoniêto et al., 2016). Similarly, our results pointed to the downregulation of amino acid transporters in sophorose-grown cultures in the Δ*xyr1* mutant strain, as well as in cellulose. At this time, the physiological consequences of these changes are unknown. Lichius et al. (2014) showed that the nuclear import of XYR1 is dependent of *de novo* protein biosynthesis, and the inhibition of protein biosynthesis abolished the entrance of XYR1 into the nucleus. These results suggest that XYR1 affects processes involved with the biosynthesis of protein in *T. reesei*. In this regard, the downregulation of amino acid transporters under cellulose and sophorose induction conditions might be explained by both a decrease in intracellular protein biosynthesis and reduced amino acid metabolism in the Δ*xyr1* mutant strain. However, further studies need to be undertaken to better understand this process.

The data shown here provides evidence for the multiple roles of XYR1 in cellulase production in *T. reesei* and how fine-tuned this process can be, as it involves regulating of the entry of nutrients through the cell as well the regulation of other TFs ultimately culminating or not culminating in cellulase synthesis.

## Conclusions

Our study contributes to a better understanding of the role of the transcription factor XYR1 in the degradation of cellulosic material by *T. reesei*. Our transcriptomic analysis showed that several genes have their expression affected in a carbon source-dependent manner. The transcriptional profile of Δ*xyr1* was drastically altered during growth in the presence cellulose, sophorose, and glucose. The most differentially expressed genes include mainly cellulolytic enzymes, but expression of transporters and transcriptional factors are also affected by XYR1. Here, we highlighted the modulation of MFS and ABC family transporters. Since transport across the membrane is the first step at which nutrient supply is tightly regulated in response to intracellular needs and often also the rapidly changing external environment, the study of these transporters will contribute to the understanding of the molecular mechanisms underlying the regulation of cellulolytic enzyme synthesis in this fungus. Furthermore, these data will contribute to the construction of industrial strains of *T. reesei* that produce high levels of cellulase for plant cell-wall degradation thus facilitating its application in fungal biotechnology.

## Author contributions

Conceived and designed the experiments: RS, GP, RSR. Performed the experiments: Ld, Rd, AA. Analyzed the data: GP, RSR, LD, RP, AA. Wrote the paper: Rd, Ld, AA, RS. All authors have read and approved the final manuscript.

### Conflict of interest statement

The authors declare that the research was conducted in the absence of any commercial or financial relationships that could be construed as a potential conflict of interest.

## Supplementary material

Figure S1 **Outline of the strategy used for sequencing**.

Figure S2 **Biological replicates used for the RNA-seq analysis (QM9414). (A–G)** Graphs representing the Pearson correlation between biological replicates of each sample demonstrating the reliability of RNA-seq (*R* ≥ 0.71). Sample QMGlu2 was removed from subsequent analysis as it was considered not informative.

Figure S3 **Biological replicates used for the RNA-seq analysis (Δ***xyr1***). (A–I)** Graphs representing the Pearson correlation between biological replicates of each sample demonstrating the reliability of RNA-seq (*R* ≥ 0.67).

Figure S4 **Principal-components analysis (PCA) of samples analyzed. (A)** Principal component analysis of the samples analysed for assessing overall similarity between the conditions using DESeq2's plotPCA. **(B)** Sample QMGlu2 was removed from downstream analysis.

Figure S5 **Box plot of all samples pre- and post-normalization. (A)** Raw counts and **(B)** Normalized counts.

Figure S6 **Differentially expressed genes (DEG) in Δ***xyr1*** compared to parental strain QM9414 grown in cellulose, sophorose, and glucose as sole carbon sources. (A)** Δ*xyr1*/QM9414 Cellulose, **(B)** Δ*xyr1*/QM9414 Sophorose, and **(C)** Δ*xyr1*/QM9414 Glucose. Differentially, expressed genes identified by DESeq2 package are plotted in red (*p* ≤ 0.05). **(D–F)** Volcano plot of differentially expressed genes under all conditions studied. Log_2_ fold changes and their corresponding −log 10 *p*-values of all genes in the RNA-seq are shown. Up-regulated genes (log_2_ fold change ≥1 and *p* ≤ 0.05) are depicted in red and down regulated (log_2_ fold change ≤ −1 and *p* ≤ 0.05) in green.

Figure S7 **Person correlation of estimated transcript levels measured by RNA-seq and qRT-PCR results**. Comparison between gene expression values obtained by RNA-seq and qRT–PCR was performed using 20 genes (Table S13). Real- time PCR was performed using the amplified cDNA from each RNA-seq sample. The solid line and R were generated by linear regression analysis using GraphPad Prism Version 5.0. A high and statistically significant Pearson correlation between the expression levels measured using real-time PCR and RNA-seq was obtained (*R* = 0.81, *P* < 0.0001). Conditions used for validation: cellulose and sophorose.

Table S1 **qRT-PCR primers used in this study**.

Table S2 **Summary of RNA-seq reads obtained for the parental strain QM9414**.

Table S3 **Summary of RNA-seq reads obtained for Δ***xyr1*** mutant strain**.

Table S4 **Top 15 down-regulated genes in the mutant Δ***xyr1*** in cellulose, sophorose and glucose**.

Table S5 *****T. reesei*** CAZymes analyzed in this study**.

Table S6 *****T. reesei*** CAZymes identified exclusively in cellulose condition**.

Table S7 *****T. reesei*** CAZymes identified exclusively in sophorose condition**.

Table S8 *****T. reesei*** CAZymes identified exclusively in glucose condition**.

Table S9 **Direct regulation by XYR1 on CAZymes identified in each condition studied**.

Table S10 **Transcriptional factors differentially modulated in each condition studied**.

Table S11 **Direct regulation by XYR1 in inducing conditions**.

Table S12 **Transporter genes differentially modulated in each condition studied**.

Table S13 **Comparison of gene expression levels assayed by RNA-seq and qRT-PCR**.
