# Supplementary figures and images for: Understanding the Role of the Master Regulator XYR1 in Trichoderma reesei by Global Transcriptional Analysis
Source: Front Microbiol. 2016 Feb 16;7:175. doi: 10.3389/fmicb.2016.00175 (PMC4754417; doi:10.3389/fmicb.2016.00175)

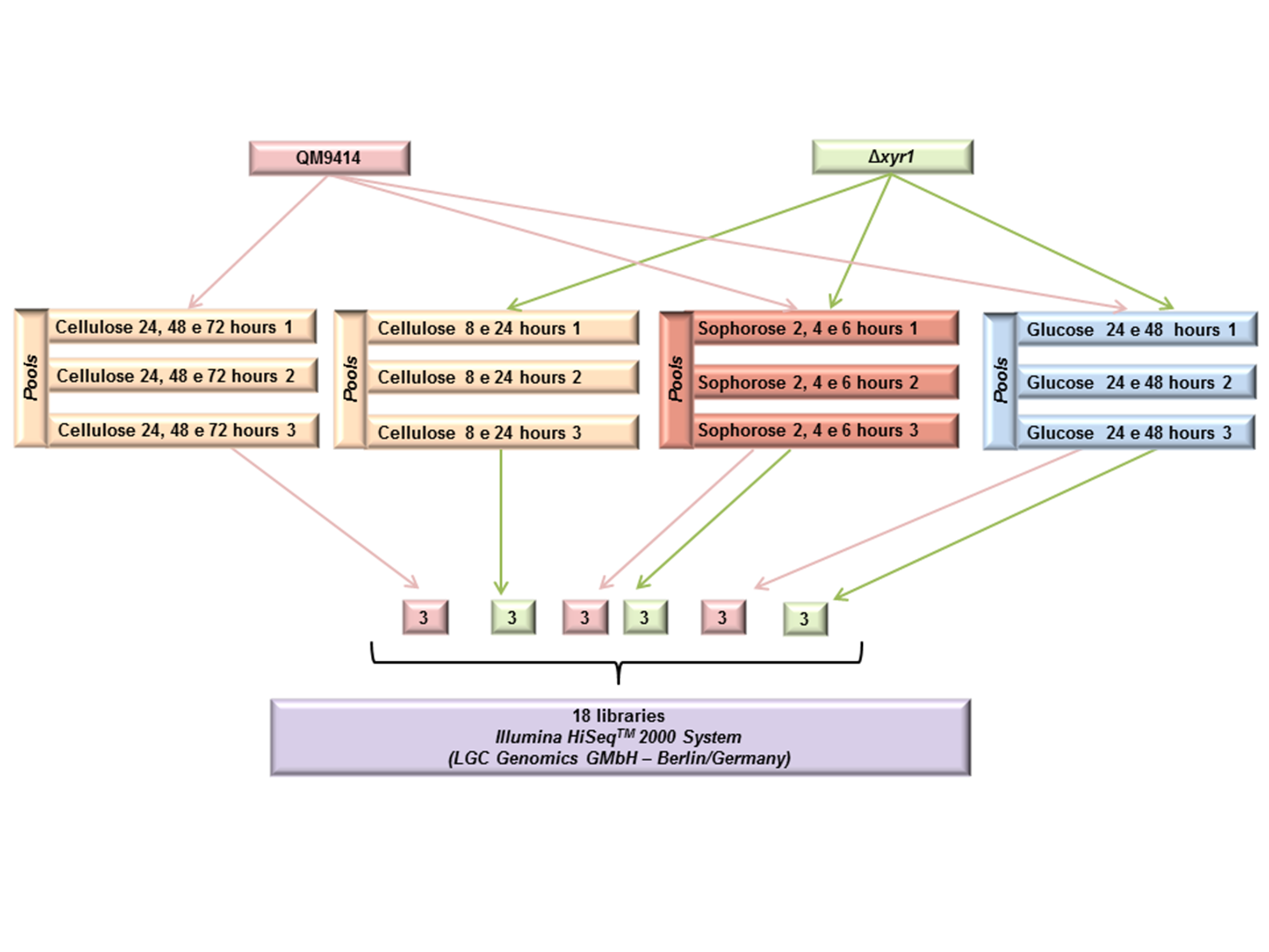

Supplement: Figure S1 — Outline of the strategy used for sequencing. [file Image1.TIF]

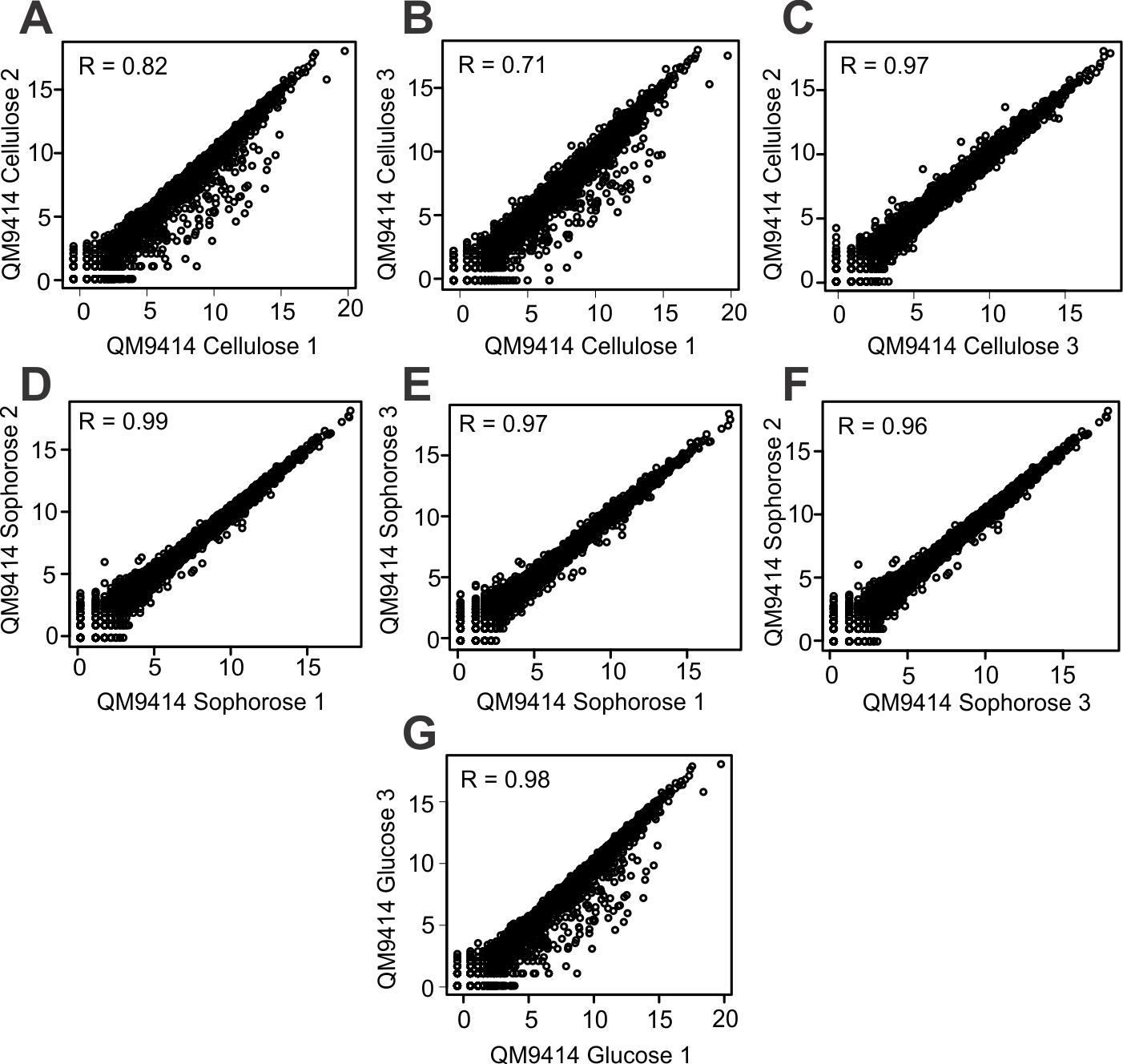

Supplement: Figure S2 — Biological replicates used for the RNA-seq analysis (QM9414). (A–G) Graphs representing the Pearson correlation between biological replicates of each sample demonstrating the reliability of RNA-seq (R ≥ 0.71). Sample QMGlu2 was removed from subsequent analysis as it was considered not informative. [file Image2.TIF]

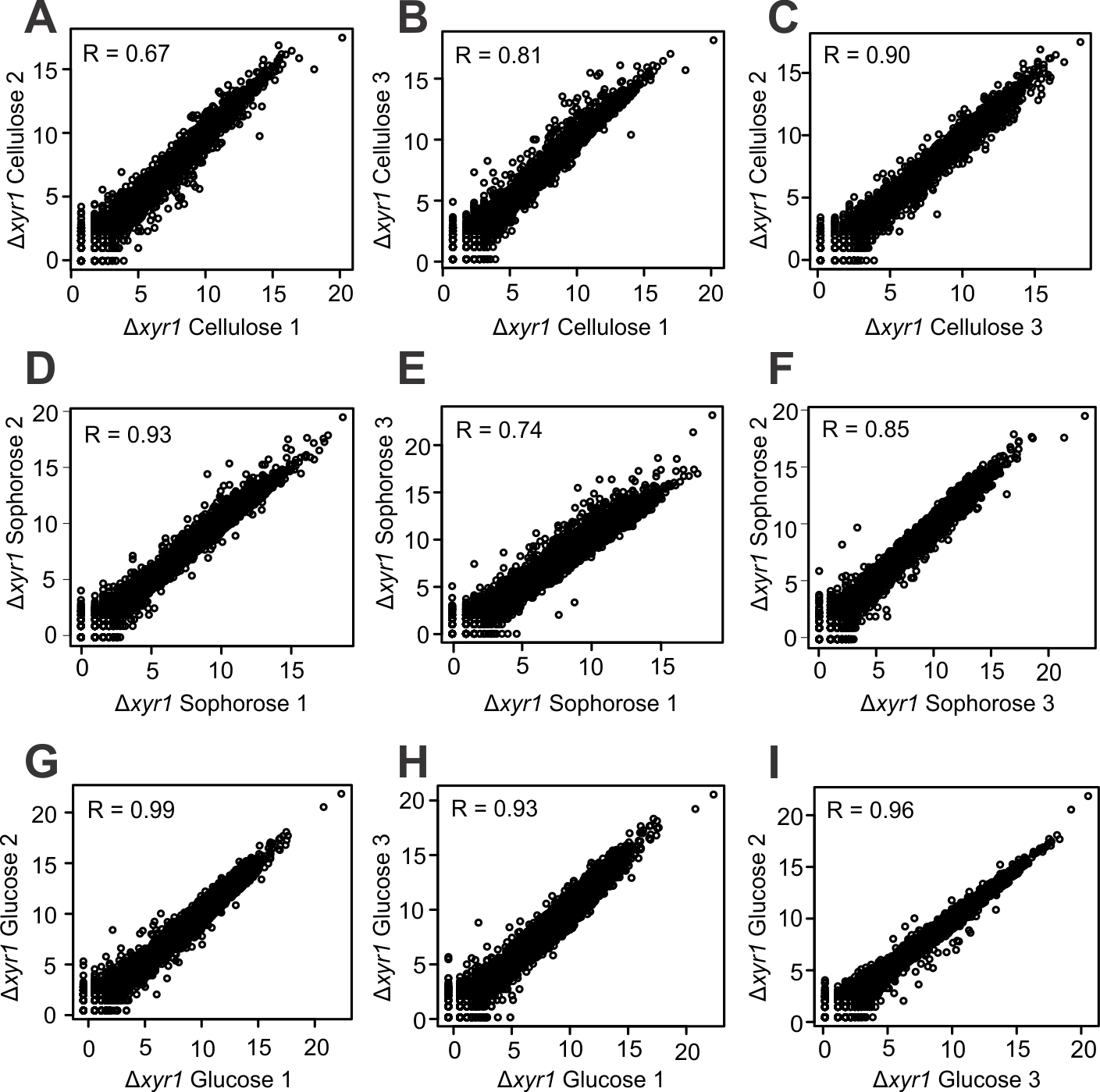

Supplement: Figure S3 — Biological replicates used for the RNA-seq analysis (Δxyr1). (A–I) Graphs representing the Pearson correlation between biological replicates of each sample demonstrating the reliability of RNA-seq (R ≥ 0.67). [file Image3.TIF]

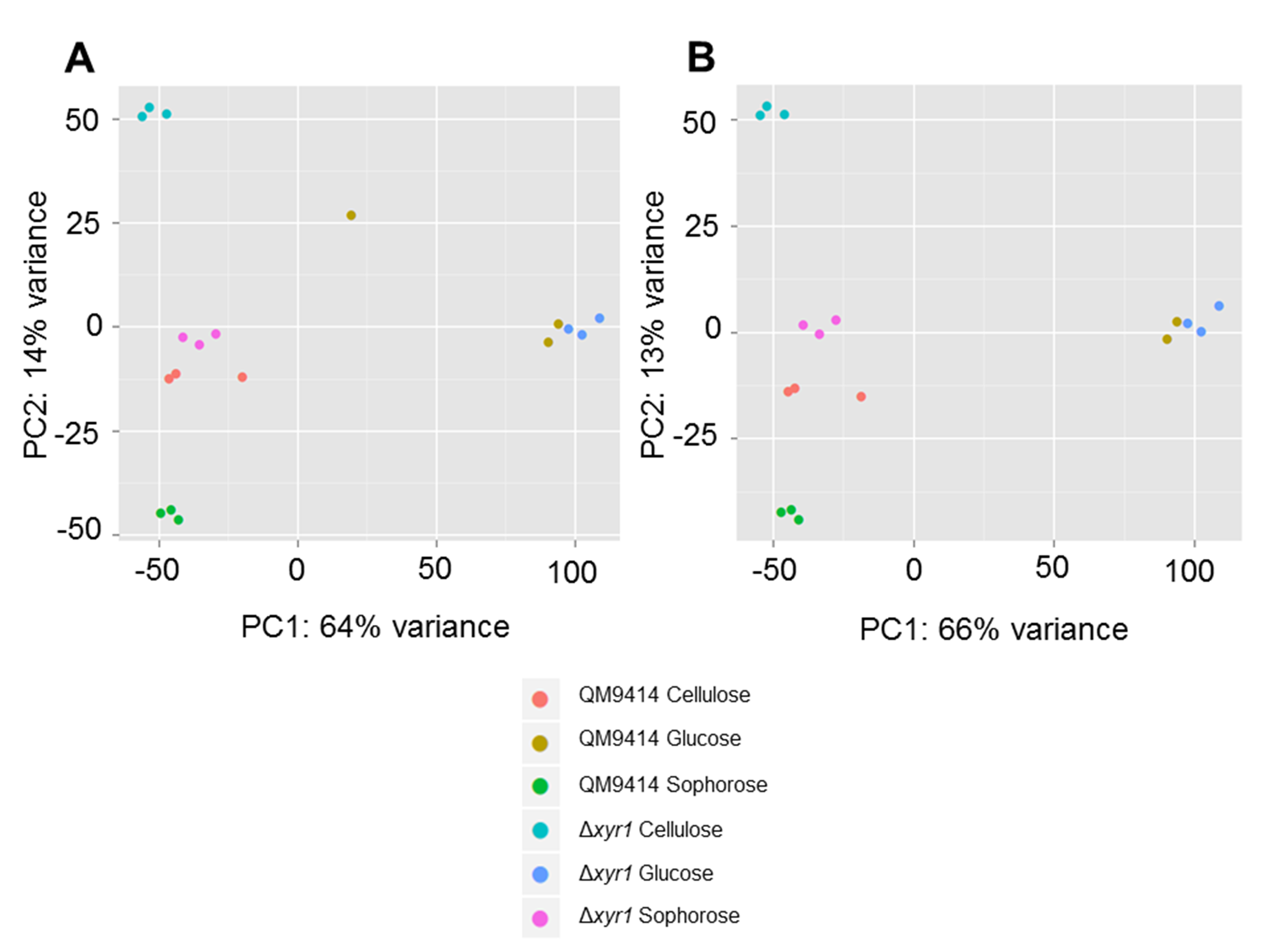

Supplement: Figure S4 — Principal-components analysis (PCA) of samples analyzed. (A) Principal component analysis of the samples analysed for assessing overall similarity between the conditions using DESeq2's plotPCA. (B) Sample QMGlu2 was removed from downstream analysis. [file Image4.TIF]

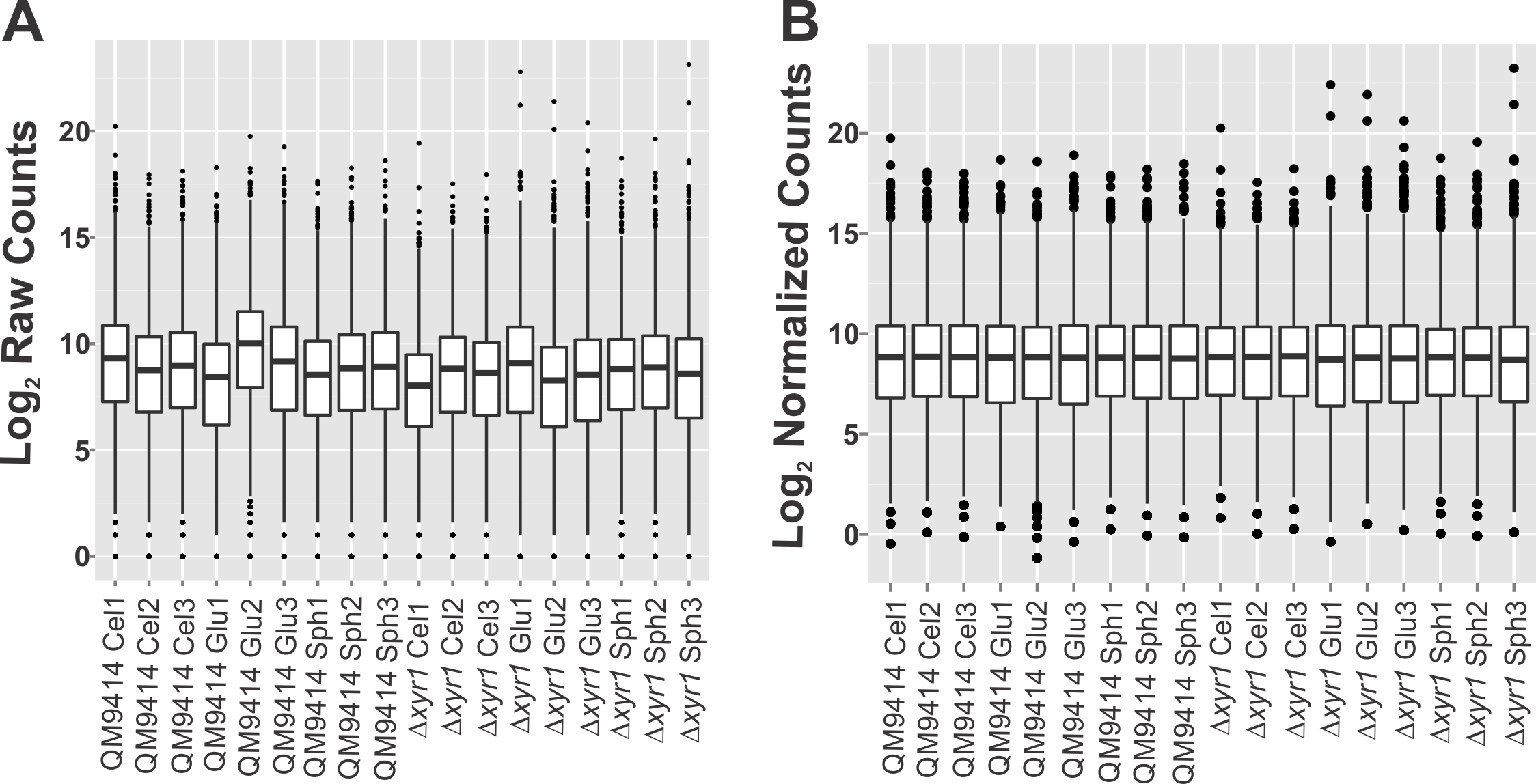

Supplement: Figure S5 — Box plot of all samples pre- and post-normalization. (A) Raw counts and (B) Normalized counts. [file Image5.TIF]

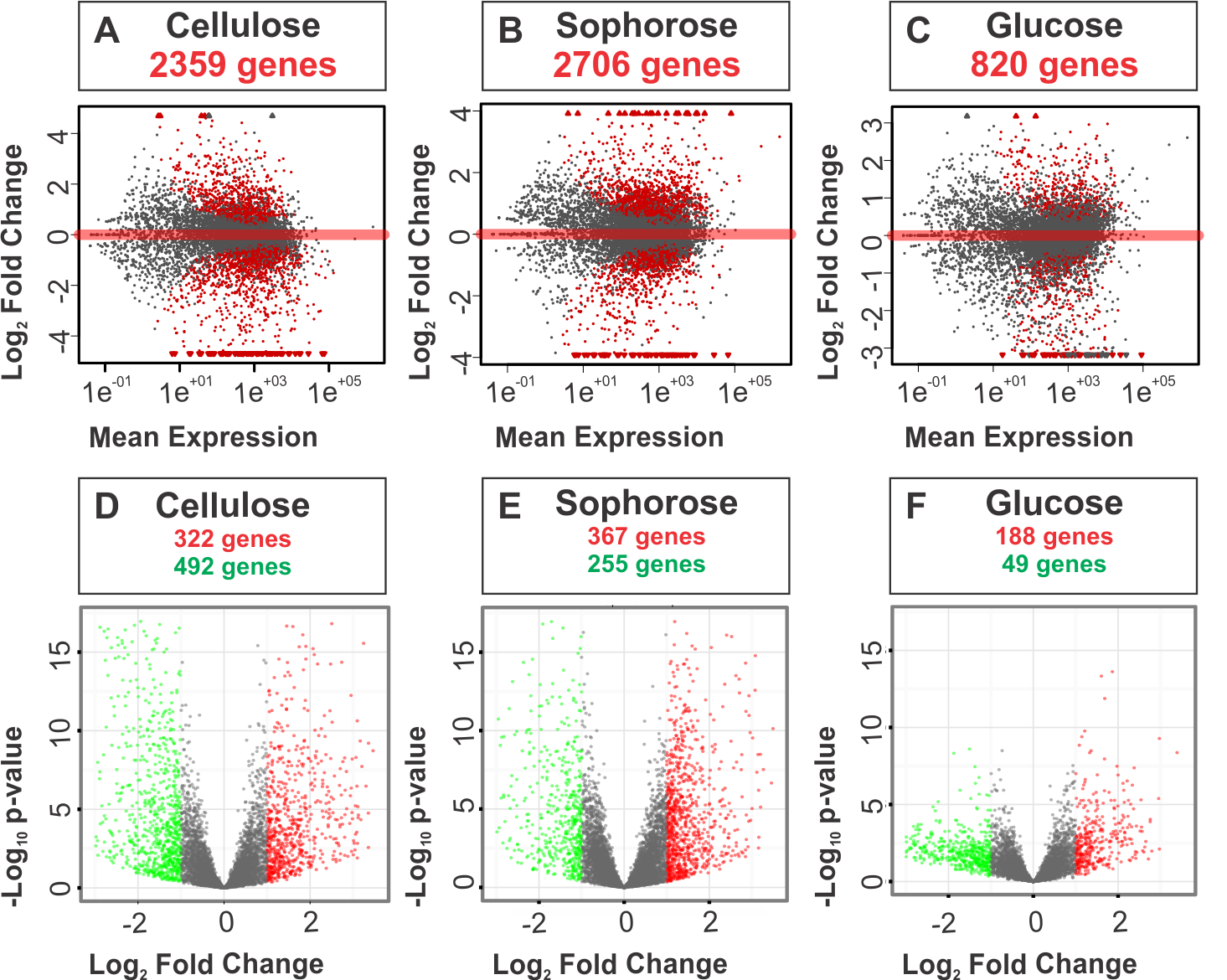

Supplement: Figure S6 — Differentially expressed genes (DEG) in Δxyr1 compared to parental strain QM9414 grown in cellulose, sophorose, and glucose as sole carbon sources. (A) Δxyr1/QM9414 Cellulose, (B) Δxyr1/QM9414 Sophorose, and (C) Δxyr1/QM9414 Glucose. Differentially, expressed genes identified by DESeq2 package are plotted in red (p ≤ 0.05). (D–F) Volcano plot of differentially expressed genes under all conditions studied. Log2 fold changes and their corresponding −log 10 p-values of all genes in the RNA-seq are shown. Up-regulated genes (log2 fold change ≥1 and p ≤ 0.05) are depicted in red and down regulated (log2 fold change ≤ −1 and p ≤ 0.05) in green. [file Image6.TIF]

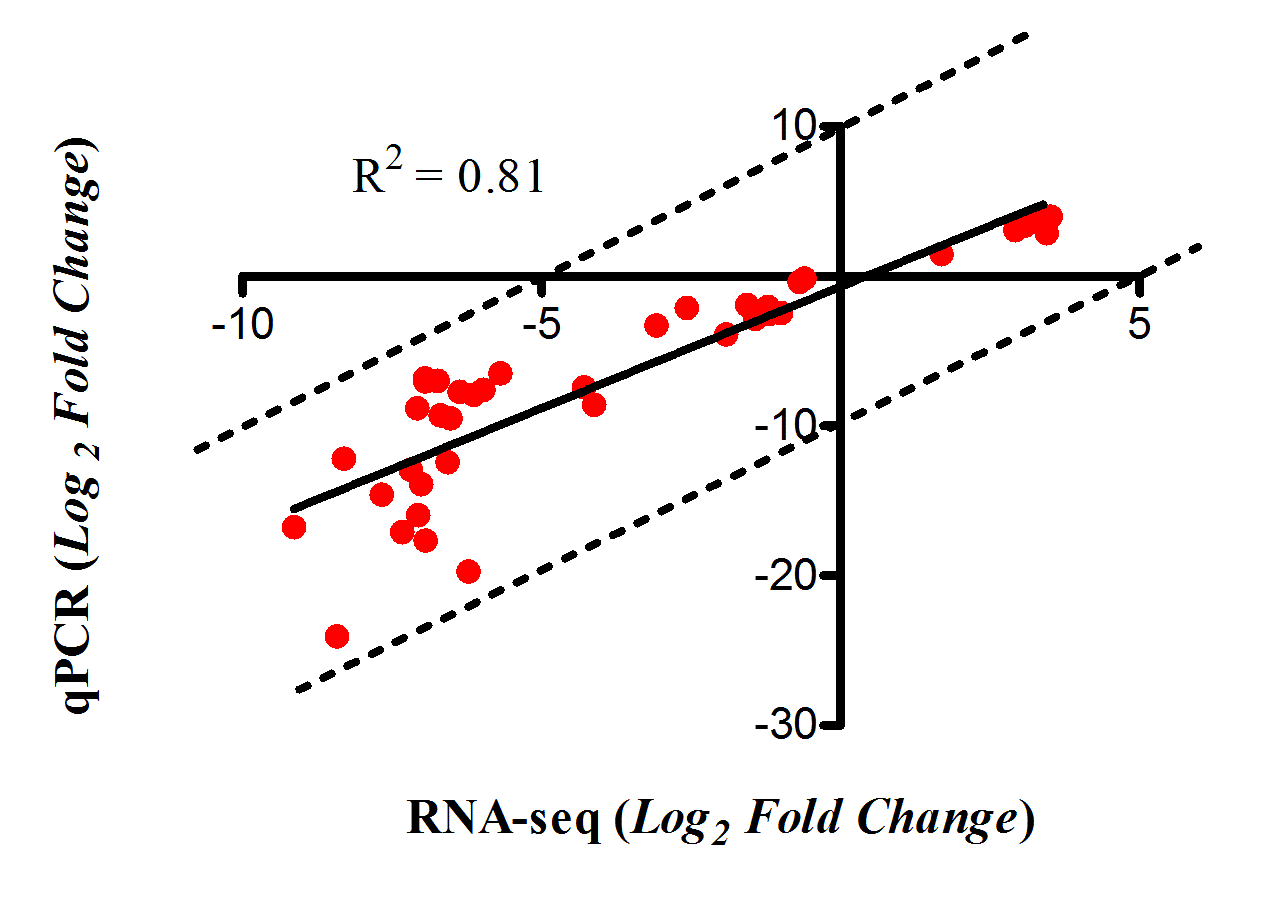

Supplement: Figure S7 — Person correlation of estimated transcript levels measured by RNA-seq and qRT-PCR results. Comparison between gene expression values obtained by RNA-seq and qRT–PCR was performed using 20 genes (Table S13). Real- time PCR was performed using the amplified cDNA from each RNA-seq sample. The solid line and R were generated by linear regression analysis using GraphPad Prism Version 5.0. A high and statistically significant Pearson correlation between the expression levels measured using real-time PCR and RNA-seq was obtained (R = 0.81, P < 0.0001). Conditions used for validation: cellulose and sophorose. [file Image7.TIF]
